# Marine biodiversity baseline for Área de Conservación Guanacaste, Costa Rica: published records

**DOI:** 10.3897/zookeys.652.10427

**Published:** 2017-02-06

**Authors:** Jorge Cortés

**Affiliations:** 1 Centro de Investigación en Ciencias del Mar y Limnología (CIMAR), Universidad de Costa Rica, San Pedro, 11501 San José, Costa Rica; 2 Escuela de Biología, Universidad de Costa Rica

**Keywords:** Marine organisms, marine ecosystems, marine biodiversity, conservation areas, Central America, compilation

## Abstract

The diversity of tropical marine organisms has not been studied as intensively as the terrestrial biota worldwide. Additionally, marine biodiversity research in the tropics lags behind other regions. The 43,000 ha Sector Marino of Área de Conservación Guanacaste (ACG, Marine Sector of Guanacaste Conservation Area), on the North Pacific coast of Costa Rica is no exception. For more than four decades, the terrestrial flora and fauna has been studied continuously. The ACG marine biodiversity was studied in the 1930’s by expeditions that passed through the area, but not much until the 1990’s, except for the marine turtles. In the mid 1990’s the Center for Research in Marine Science and Limnology (CIMAR) of the Universidad de Costa Rica (UCR) initiated the exploration of the marine environments and organisms of ACG. In 2015, ACG, in collaboration with CIMAR, started the BioMar project whose goal is to inventory the species of the marine sector of ACG (BioMar ACG project). As a baseline, here I have compiled the published records of marine ACG species, and found that 594 marine species have been reported, representing 15.5% of the known species of the Pacific coast of Costa Rica. The most diverse groups were the crustaceans, mollusks and cnidarians comprising 71.7% of the ACG species. Some taxa, such as mangroves and fish parasites are well represented in ACG when compared to the rest of the Costa Rican coast but others appear to be greatly underrepresented, for example, red algae, polychaetes, copepods, equinoderms, and marine fishes and birds, which could be due to sampling bias. Thirty species have been originally described with specimens from ACG, and 89 species are not known from other localities on the Pacific coast of Costa Rica except ACG. Most of the sampling has been concentrated in a few localities in Sector Marino, Playa Blanca and Islas Murciélago, and in the nearby waters of Bahía Santa Elena. In an effort to fill this gap, CIMAR is collaborating with ACG and a private foundation to start an inventory of the marine organisms of the conservation area. The project will be assisted by two marine parataxonomists, and all samples will be catalogued, photographed, bar coded and voucher specimens deposited at the Museo de Zoología, UCR. All the information will be available through Internet. It is anticipated that the BioMar project will fill many of the knowledge gaps and significantly more marine species will be encountered. This project could become a viable model for marine biodiversity inventories in other Costa Rican Conservation Areas (Áreas de Conservación) and in other countries.

## Introduction

Marine biodiversity studies have lagged behind terrestrial research, especially in the tropics, with a few exceptions such as Australia (Chapman 2009). Some studies in the Neotropics regarding marine biodiversity have been published, most focused on coral reef areas (Cortés et al. 2017). Several taxonomic groups are fairly well known, such as mollusks and fishes, with monographs, many papers and guides, while others are poorly known, to mention a few, microorganisms and smaller phyla. The same occurs geographically: some countries in the tropics have been relative well studied, for example, Costa Rica (Wehrtmann and Cortés 2009), while in other countries (such as Nicaragua) research and publications on marine biodiversity are scarce.

**Figure 1.**
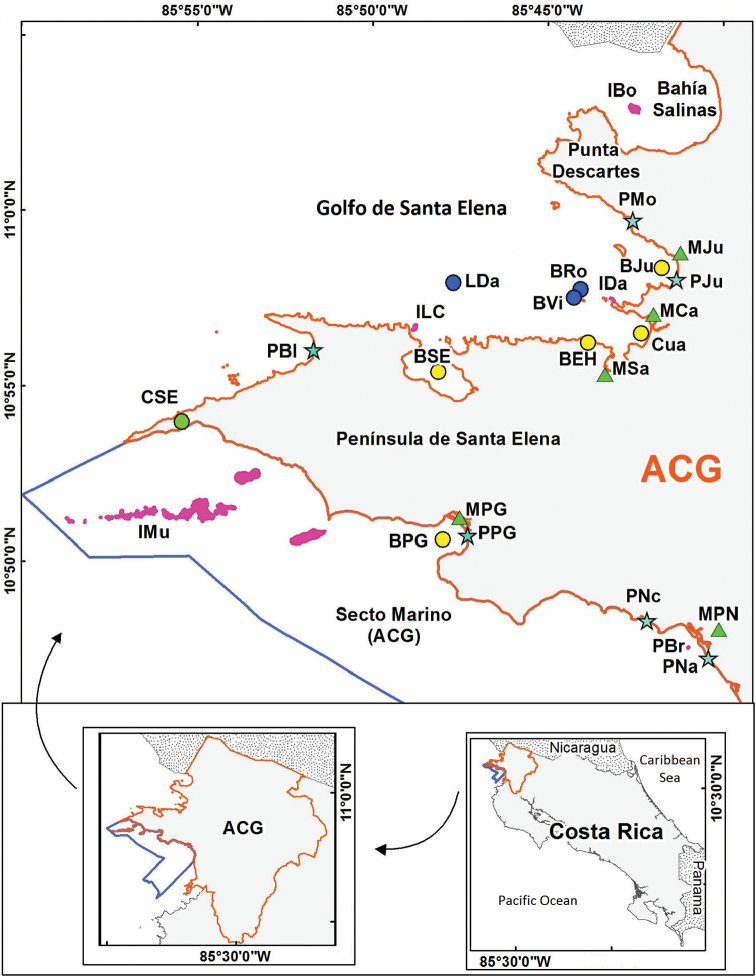
Map of the Área de Conservación Guanacaste (ACG) in the northern Pacific coast of Costa Rica with indication of the sites mentioned in the text. See Table 2 for the codes of the sites. Stars = beaches, triangle = mangrove forests, circle = bays; green = protected area; blue circles = shoals.

Costa Rica comprises 11 Conservation Areas (Áreas de Conservación), one of which is Área de Conservación Guanacaste (ACG) on the northwest Pacific coast of Costa Rica (Fig. 1). The ACG contains much of the last remnants of Costa Rican tropical dry forest and its terrestrial biodiversity has been and still is the subject of intensive research and restoration (Janzen and Hallwachs 2016). The ACG covers an area of 163000 hectares, 43000 of them marine, and 150 km of protected coastline (http://www.acguanacaste.ac.cr/acg/que-es-el-acg). It was declared a UNESCO World Heritage Natural Site in 1999. Compared to the terrestrial area, the marine sector (officially Sector Marino) has not been studied intensively. A new initiative, BioMar ACG (Marine Biodiversity of ACG), was started in 2015 to inventory the marine organisms of the area, and then make all the information publicly available, mainly through the Internet, but also with scientific and popular publications. This project is a 5-year collaboration between the conservation area, a private foundation and academia; all samples are being catalogued, photographed, bar coded, and vouchers deposited at the Museo de Zoología (Museum of Zoology) at the Universidad de Costa Rica (UCR).

The marine sector of ACG has a high diversity of habitats, with high species richness worthy of more study (Beebe 1942, Cortés 1996–1997b). There is a well-represented suite of coastal and marine ecosystems, such as mangrove forest of variable sizes, beaches of different composition and size, bays and coves, rocky intertidal zones with several wave regimens, mud flats, rocky subtidal sites, coral reefs, rhodolith beds and deep areas – more than 50 m, plus an archipelago (Islas Murciélago), shoals, and several more isolated islands (Cortés and Wehrtmann 2009, Cortés 2016). The main nesting site in the country of the frigate bird, *Fregata magnificens*, is on one of the nearby islands, Isla Bolaños, in Bahía Salinas (Alvarado-Quesada 2006). An outstanding oceanographic feature of the region is the seasonal upwelling (the Papagayo Upwelling) that brings deep cold, nutrient-rich and CO_2_-rich waters to the surface during the trade winds season (December to April-May) (McCreary et al. 1989, Alfaro and Cortés 2012, Rixen et al. 2012). Micro- and macroalgal growth increases significantly as a consequence of the upwelling (Cortés et al. 2014).

What is now ACG’s Sector Marino (Fig. 1) was first explored, samples collected, and papers published by several marine expeditions from the United States starting in the 1930’s (Cortés 2009a, Table 1). The first expedition was the Templeton Crocker Expedition of the California Academy of Sciences in 1932 aboard the SY *Zaca*, when they visited Bahía Murciélago and Bahía de Santa Elena (previously known as Port Parker) (Crocker 1933). In 1935, as part of the Allan Hancock Pacific Expeditions aboard the MY *Velero III*, biologists visited Bahía Santa Elena and Bahía Salinas (Fraser 1943a, b). The SY *Zaca* was again in the region in 1937–1938, but this time in an expedition of the New York Zoological Society; they collected in Bahía Santa Elena, around Islas Murciélago and around Playa Potrero Grande (Beebe 1938, 1942). These three expeditions generated a significant number of publications on ACG marine organisms (Table 1). There were no additional expeditions until 1959, when the MY *Stella Polaris* visited the country (Dawson and Beaudette 1959). In 1972, the RV *Searcher* collected samples in the region and new species of fish were described (Bussing and Lavenberg 2003). The next expedition that visited the area was the Eastern Pacific RV *Alpha Helix* Expedition, in 1978 organized by the Scripps Institution of Oceanography (SIO). They collected samples that are deposited at SIO, but few papers were published (Luke 1995). Chan et al. (2016) recently published on some of the barnacles collected during that expedition. The most recent expedition was the Smithsonian Tropical Research Institute RV *Urracá* to the northern and central Pacific coast of Costa Rica in 2005 (Vargas-Castillo 2008).

**Table 1.** Historical account of marine studies at the Área de Conservación Guanacaste, Pacific coast of Costa Rica.

Years	Expedition/Project/Institutions/Individual	Taxon/System	References
1932	The Templeton Crocker Expedition of the California Academy of Sciences, aboard the SY *Zaca*	Algae and mollusks	93, 104, 184
1935	The Allan Hancock Pacific Expeditions, aboard the MY *Velero III*	Foraminifera, corals, hydroids, mollusks, crustaceans and echinoderms	5, 28, 50, 51, 52, 53, 54, 62, 63, 68, 76, 77, 78, 79, 90, 94, 95, 96, 119, 127, 148, 152, 156, 157, 158, 180, 186, 188, 203
1937–1938	Eastern Pacific Expeditions of the New York Zoological Society, aboard the SY *Zaca*	Mollusks, crustaceans and echinoderms	44, 45, 46, 47, 61, 80, 81, 82, 91, 105, 106, 107, 108, 109, 110, 111, 112, 113, 114, 136, 189
1959	Eastern Pacific cruise, aboard the MY *Stella Polaris*	Algae	56, 57, 58
1970 -present	Many individuals, for example, SE Cornelius, LG Fonseca, DA Hughes, JD Richard, DC Robinson, JR Spotila and RA Valverde	Turtle studies	1, 10, 25, 33, 34, 35, 36, 37, 48, 49, 65, 66, 67, 70, 71, 74, 84, 89, 115, 120, 121, 122, 123, 147, 150, 153, 155, 164, 165, 166, 167, 168, 169, 175, 183, 190, 192, 193, 202
1972	Central American Expedition/Janss Foundation, aboard the RV *Searcher*	Crustacean and fish	21
1973 -present	Several individuals and groups, e.g. DJ Pool, FE Putz and CIMAR, UCR	Mangroves	128, 170, 172, 208
1978	Caribbean-Pacific Expedition Phase VI/ Scripps Institution of Oceanography, aboard the RV *Alpha Helix*	Mollusks and crustaceans	27, 129, 130
1984 -present	CIMAR, UCR	Coral reefs	7, 39, 42, 124
1984 -present	CIMAR, UCR	Octocorals, corals, anemones, crustaceans, fishes, marine mammals,	11, 12, 13, 14, 15, 16, 17, 18, 19, 20, 38, 41, 59, 60, 64, 72, 141, 142, 143, 144, 145, 151, 160, 187
1991, 2013, present	Museo de Zoología, UCR	Crustaceans	102, 194, 195, 196, 197, 199, 201
1996, 1998	Fish parasite studies	Platyhelmiths and acathocephalans	24, 138, 149, 159
1996, 2002	Instituto Nacional de Biodiversidad	Mollusks	22, 23, 133, 191
2005	Benthic survey of northern and central Costa Rica/Smithsonian Tropical Research Institute, aboard the RV *Urracá*	Crustaceans	198
2005	Museo Nacional de Costa Rica	Birds	2
2006, 2011	Universidad Nacional, Heredia	Ascidians and cetaceans	139, 154
2014 -present	CIMAR, UCR	Beaches and rocky shores	185

Many individuals, groups of researchers or institutions have contributed to the knowledge of ACG marine biodiversity (Table 1). Elmer Y. Dawson published several papers on macroalgae of Costa Rica, including the ACG (Dawson 1960, 1961). Richard and Hughes (1972) and Cornelius (1975) published on marine turtles of the ACG, with the first observations in 1970–1971. In 1996, Marques et al. (1997) and Monks et al. (1997) collected and later described several fish parasites. Between 1996 and 2002, the Instituo Nacional de Biodiversidad collected mollusks in the ACG, and generated several papers on the opistobranchs (Valdés and Camacho-García 2004, Camacho-García et al. 2005, Camacho-García and Gosliner 2008). The CIMAR of the UCR has published papers on marine organisms and environments of Costa Rica that include the ACG: e.g., Cutler et al. (1992) on sipunculids, Moran and Dittel (1993) - crustaceans, Cortés and Guzmán (1998) - corals, Dean (2001, 2004) - polychaetes, Suárez-Morales and Morales-Ramírez (2001) - copepods, and Heard et al. (2009) - tanaidaceans. Also, new species have been described from the ACG: a crustacean (Vargas 2000), two octocorals (Breedy and Guzman 2003) and a fish (Del Moral-Flores et al. 2015). Cortés and Jiménez (2003) provided a description of the coral reefs of the ACG, while Loría-Naranjo et al. (2014) evaluated the main mangrove forests and Sibaja-Cordero et al. (2014) the beach fauna. Even so, our knowledge about the species diversity of the ACG is far from complete.

The objective of this contribution is to generate a baseline of the marine biodiversity of ACG’s Sector Marino and adjacent unprotected areas, some of which are in the process of being officially protected. This will serve as a starting point for the recently initiated BioMar ACG project (Marine Biodiversity of the Guanacaste Conservation Area). This five-year project (2015–2019), funded by the Guanacaste Dry Forest Conservation Fund, and with support from the Ministry of the Environment and Energy of the Costa Rican government and the UCR, will collect, identify and provide publicly accessible information about most of ACG’s species of marine macroorganisms and as many of the microorganisms as feasible.

## Materials and methods

The study area is Sector Marino of the ACG and adjacent areas, located on the North Pacific of Costa Rica (Fig. 1, Table 2). Publications about ACG marine organisms were compiled and analyzed. A list of recorded species was created based on those publications. Later all scientific names were updated using WoRMS (World Register of Marine Species, http://www.marinespecies.org/), AlgaeBase, http://www.algaebase.org (Guiry and Guiry 2016), Encyclopedia of Life (http://eol.org/), Bryozone (http://bryozone.myspecies.info/), Integrated Digitized Biocollections (https://www.idigbio.org/), Worldwide Mollusc Species Data Base (http://www.bagniliggia.it/WMSD/Lindex_aaa.htm), SeaLifeBase (http://www.sealifebase.org/) and ZipcodeZoo (http://zipcodezoo.com/index.php/Main_Page).

**Table 2.** Localities of the samples reported in the Appendix 1. # spp. = number of species reported from that site. a = Protected area, b = area in the process of being officially protected, c = marine area not protected, and d = private reserve (protected area).

Code	Locality / area	Notes	# spp.
ACG ^a^	Área de Conservación Guanacaste	Entire Conservation Area	13
BEH^c^	Bahía El Hachal	Bay	6
BJu^c^	Bahía Junquillal	Bay	5
BPG^a^	Bahía Potrero Grande	Bay	18
BRo^c^	Bajo Rojo	Shoal	2
BSE^b^	Bahía Santa Elena	Bay	371
BVi^c^	Bajo Viejón	Shoal	5
CSE^a^	Cabo Santa Elena	Tip of PSE	23
Cua^c^	Cuajiniquil	Off Cuajiniquil	6
IBo^c^	Isla Bolaños	Island	1
IDa^c^	Isla David	Island	7
ILC^b^	Isla Los Cabros	Island	1
IMu^a^	Islas Murciélago	Archipelago	103
Jun^a^	Junquillal	Off Junquillal	21
LDa^c^	La Danta	Shoal	1
MCa^a^	Manglar de Cuajiniquil	Mangrove forest	14
MJu^a^	Manglar de Junquillal	Mangrove forest	6
MPG^a^	Manglar de Potrero Grande	Mangrove forest	14
MPN^a^	Manglar de Playa Naranjo	Mangrove forest	19
MSa^a^	Manglar Salinita	Mangrove forest	14
PBl^a^	Playa Blanca	Beach	104
PBr^a^	Peña Bruja	Islet off PNa	2
PPG^a^	Playa de Potrero Grande	Beach	4
PJu^a^	Playa Junquillal	Beach	2
PMo^d^	Playa Mostrencal	Beach	3
PNa^a^	Playa Naranjo	Beach	10
PNc^a^	Playa Nancite	Beach	16
PSE^a^	Península de Santa Elena	Peninsula	12
SMa^a^	Sector Marino ACG	Marine Sector of ACG	4

The resulting list of species was compared to the remainder of the Pacific coast of Costa Rica and to available species lists from other countries in the Eastern Tropical Pacific. Knowledge gaps were identified and potential areas of future research suggested.

## Results

Five hundred ninety four marine species have been reported so far for the ACG (Table 3, Appendix 1), which represents 15.5% of the known species of the Pacific coast of Costa Rica. The most diverse groups were crustaceans (193 spp.), mollusks (187 spp.) and cnidarians (46 spp.), comprising together 71.7% of the ACG’s marine species. These three groups represent 23.9%, 18.2% and 26.7%, respectively of the known species of the Pacific coast of the country (Table 3). Some groups are well represented in the ACG when compared to the rest of the coast (e.g., species of mangroves and fish parasites), while others are greatly underrepresented. For example, red algae, polychaetes, copepods, equinoderms, and marine fishes and birds are poorly represented in the published reports (Table 3). Other groups of organisms have been observed and identified (e.g., various species of sponges, flat worms, ophiuroids, and ascidian) but there are no published records of these species (Table 4). Other taxa (such as diatoms, nemerteans and appendicularians) undoubtedly inhabit the study area but have not been observed or collected yet (Table 4).

**Table 3.** Number of marine species reported from Área de Conservación Guanacaste (complete list of species in the Appendix 1), Pacific coast of Costa Rica (see Cortés 2012, plus references indicated as superindex) (species reported only for Isla del Coco were excluded); percentage of the species of the Pacific reported form ACG, and species only found in ACG. n.k. = not known.

TAXA	Species from ACG	Species from Pacific Costa Rica	% of species of the Pacific	Species only at ACG
Bacteria	15	>17 ^103, 183^	88.2	15
Cyanobacteria	4	28	14.3	2
Chlorophyta	4	44 ^73^	9.1	2
Phaeophyceae	6	26 ^73^	23.1	1
Rhodophyta	15	146 ^73^	10.3	9
Mangroves	7	8	87.5	0
Foraminifera	24	76	31.6	12
Cnidaria	46	172	26.7	2
Anthozoa	35	59	59.3	2
Hydrozoa	11	108	10.2	0
Platyhelminthes	7	38 ^40, 178^	18.4	7
Trematoda	4	20 ^40,178, 182^	20.0	4
Cestoda	3	12 ^40, 178^	25.0	3
Acanthocephala	1	1 ^149^	100	0
Mollusca	187	1025	18.2	0
Gastropoda	85	631	13.5	0
Bivalvia	102	362	28.2	0
Sipuncula	3	15	20.0	0
Annelida	24	313	7.7	11
Nemertea	1	Several species	n.k.	n.k.
Crustacea	193	807	23.9	13
Amphipoda	13	106	12.3	8
Cumacea	1	19 ^161^	5.3	1
Decapoda	162	409	39.6	1
Mysida	1	5	20.0	0
Stomatopoda	10	27	37.0	0
Tanaidacea	1	5	20.0	1
Copepoda	1	163	0.61	1
Cirripedia	4	36	11.1	1
Bryozoa	9	39	23.1	8
Echinodermata	15	105	14.3	7
Asteroidea	1	12	8.3	0
Echinoidea	1	28	3.6	0
Holothuroidea	13	28	46.4	7
Chordata	33	961	3.4	0
Ascidiacea	5	14	35.7	0
Cephalochordata	1	2	50	0
Elasmobranchii	3	68	4.4	0
Actinopterygii	11	774	1.4	0
Reptilia	4	5	80.0	0
Aves	2	76	2.6	0
Mammalia	7	22	31.8	0
TOTAL	594	3821+	15.5	89

**Table 4.** Taxa reported from other sites of Pacific Costa Rica (see Cortés 2012, plus references indicated as superindex), but not from Área de Conservación Guanacaste. n.k. = not known; Present = have been observed or collected but there are no publications; Probably = there is a high probability that they are present but have not been observed yet.

Taxonomic group	Number of species reported	ACG
Diatoms	174 ^131,132,200^	Present
Dinoflagellates	102	Present
Marine fungi	5 genera	n.k.
Seagrasses	2	n.k.
Porifera	62	Present
Pennatulaceans	4	Present
Scyphozoans	10	Present
Polyplacophorans	24	Present
Cephalopods	20	Present
Echiurians	1	Present
Monogeneans	10 ^40^	Probably
Nemerteans	Several species	Probably
Kinorhynchans	2	n.k.
Euphausiids	20	Present
Isopods	37	Present
Branchiopods	1	n.k.
Ostracods	2	Probably
Pycnogonids	10	Probably
Marine insects	9	Probably
Chaetognaths	27	Present
Brachiopods	8	n.k.
Phoronids	1	n.k.
Crinoids	2	n.k.
Ophiuroids	54	Present
Appendicularians	10	Probably
Thaliaceans	4	Probably
Turtle parasites	34	Present

Over 85% of the species reported are also found in other areas of the coast of Costa Rica and in the Eastern Tropical Pacific; however, most areas, including the ACG, have not been intensively collected, and the same common species are found repeatedly by collecting expeditions. Thirty new species have been described from specimens collected in the ACG: one foraminiferan, one echinoderm, two octocorals, three parasitic flatworms, four fishes, eight crustaceans and 11 mollusks (Appendix 1). Eighty-nine species are currently known only from the ACG along the Pacific coast of Costa Rica (Table 3, Appendix 1).

Most of the sampling has been concentrated in a few localities of the marine area of the ACG and those sites therefore have the highest number of reported species. For example, Bahía Santa Elena (371 spp.), Playa Blanca (104 spp.) and in some of the Islas Murciélago (103 spp.) seem very species-rich (Table 2, Appendix 1). Other areas within ACG have not been sampled at all, for example the northern shore of the Santa Elena Peninsula or some of the Islas Murciélago. The soft bottom substrate has not been sampled thoroughly nor most of the rocky intertidal zones.

## Discussion

Compared to other areas on the Pacific of Costa Rica, the ACG has fewer known marine species (594 spp.) than does Golfo Dulce (1028 spp.: Morales-Ramírez 2011) or Isla del Coco (1688 spp.: Cortés 2012), but about the same as what is currently known for Bahía Culebra (577 spp: Cortés et al. 2012). But that number will definitely increase as more taxa, other sites and environments within the ACG are inventoried.

Cortés et al. (2017) synthesized the knowledge of marine biodiversity of the Eastern Tropical Pacific, mainly from coral reefs, where most studies have been done. For example, 857 marine species have been reported for Clipperton Atoll, France, (Charpy 2009, Payri et al. 2009, Fourriére et al. 2014), 968 spp. for El Salvador (Barraza 2000, 2014a, b), 2157 spp. for the coast of Oaxaca, México (Bastida-Zavala et al. 2013), 3536 spp. for the Galápagos Islands (Bustamante et al. 2002, Hickman 2009), 3838 spp. for the Pacific coast of Costa Rica (Table 3, this paper), and 5740 spp. for the entire Gulf of California, México (Aburto-Oropeza and Balart 2001, Reyes-Bonilla et al. 2012). In other countries, for example, Panamá and Colombia, there are detailed inventories of some higher taxa, but not a compilation of all macrotaxa (Cortés et al. 2017). None of these inventories attempted to include the microorganisms.

There are large differences in the numbers of species among different sites in the Eastern Tropical Pacific and these differences could be due to several causes. First, the number, diversity and depth of research efforts influence the extent of the knowledge of the marine biodiversity of a region. Second, the extent of each region will also have an effect on species diversity, because larger areas will probably include more habitats and environments, and thus species. The ACG marine area comprises 430 km^2^, while the Gulf of California has about 160000 km^2^. Third, some sites may differ in species richness and diversity because of differences in geomorphology, oceanography, geological history and biogeography. Fourth, natural disturbances such as warming or cooling events can have a long-term impact on local biodiversity.

Knowing and documenting which species occurs where is a critical first step in understanding and conserving the biodiversity of a particular area. As outlined in Tables 3 and 4, there are important gaps in our knowledge in taxonomy and geographic distribution of marine organisms in the ACG. Much more work is needed to have an even approximately complete inventory, understand the ecological role of the species, their habitats, population structure, and distribution. Researchers of the BioMar-ACG project will fill many of these gaps, and together with other researchers from Costa Rica and elsewhere, the understanding of the marine biodiversity of the ACG will increase greatly. The BioMar project incorporates several innovative aspects, including marine parataxonomists, DNA barcoding of all organims and fast accessibility of the information. This project could serve as a viable model for marine biodiversity inventory in other Costa Rican conservation areas and in other countries.

## Appendix 1

**Table:** Marine species reported from Área de Conservación Guanacaste (ACG). Species in bold type reported only for the ACG in Costa Rica (in the case of bacteria some have been reported in people but not in marine organisms). Localities as in Figure 1 and Table 1. Localities in bold type = ^a^) Type locality, ^b^) Paratype locality and ^c^) Neotype specimen. References numbered as in the reference list.

	Species	Locality	References
Phylum ACTINOBACTERIA, Class Actinobacteria, Order Actinomycetales, Family Corynebacteriaceae	***Corynebacterium* spp.**	PNc	183
Phylum FIRMICUTES, Class Bacilli, Order Bacillales, Family Bacillaceae	***Bacillus* spp.**	PNc	183
Order Lactobacillales, Family Enterococcaceae	***Enterococcus faecalis* (Orla-Jensen, 1919) Schleifer & Kilpper-Bälz, 1984**	PNc	183
Order Lactobacillales, Family Lactobacillaceae	***Lactobacillus* spp.**	PNc	183
Order Bacillales, Family Staphylococcaceae	***Staphylococcus aureus* Rosenbach, 1884**	PNc	183
Phylum PROTEOBACTERIA, Class Betaproteobacyeria, Order Burkholderiales, Familia Alcaligenaceae	***Alcaligenes faecalis* Castellani & Chalmers, 1919**	PNc	183
Class Gammaproteobacteria, Order Aeromonadales, Family Aeromonadaceae	***Aeromonas* spp.**	PNc	183
Order Enterobacteriales, Family Enterobacteriaceae	***Citrobacter freundi* (Braak 1928) Werkman & Gillen, 1932**	PNc	183
	***Pantoea agglomerans* (Ewing & Fife, 1972) as *Enterobacter agglomerans***	PNc	183
	***Escherichia coli* Castellani & Chalmers, 1919**	PNc	183
	***Proteus mirabilis* Hauser, 1885**	PNc	183
	***Salmonella* spp.**	PNc	183
Order Pseudomonadales, Family Moraxellaceae	***Acinetobacter* spp.**	PNc	183
Order Pseudomonadales, Family Pseudomondaceae	***Pseudomonas aeruginosa* (Schroeter, 1872) Migula, 1900**	PNc	183
	***Pseudomonas* spp.**	PNc	183
Phylum CYANOBACTERIA, Class Cyanophyceae, Order Chroococcales, Family Dermocarpellaceae	***Cyanocystis violacea* (P.L. Crouan & H.M. Crouan) Komárek & Anagnostidis, 1986 as *Dermocarpa violacea***	BSE	188
Order Chroococcales, Family Entophysalidaceae	***Entophysalis granulosa* Kützing, 1843**	BSE	188
Order Oscillatoriales, Family Oscillatoriaceae	*Lyngbya semiplena* J. Agardh ex Gomont, 1892	BSE	188
Class Cyanophyceae	One species	BEH	185
Phylum CHLOROPHYTA, Class Ulvophyceae, Order Cladophorales, Family Cladophoraceae	***Cladophora lehmanniana* (Lindenberg) Kützing, 1843 as *Cladophora utriculosa***	BPG	56, 57
	*Cladophora* sp.	BSE	128
Order Ulvales, Family Ulvaceae	*Ulva flexuosa* Wulfen, 1803 as *Enteromorpha flexuosa* and as *Enteromorpha lingulata*	BSE	57, 188
	*Ulva lactuca* Linnaeus, 1753	BSE	188
	***Ulva prolifera* O.F. Müller 1778 as Enteromorpha prolifera var. flexuosa**	BSE	57, 128
	*Ulva* sp.	Jun	185
Phylum OCHROPHYTA, Class Phaeophyceae Order Dictyotales, Family Dictyotaceae	*Padina* sp.	BPG	58
Order Ectocarpales, Family Scytosiphonaceae	*Colpomenia durvillei* (Bory de Saint-Vincent) M.E. Ramírez, 1991 as *Colpomenia phaeodactyla*	BSE	207
	*Colpomenia ramosa* W.R. Taylor, 1945	BSE	57, 188
	*Colpomenia sinuosa* (Mertens ex Roth) Derbès & Solier, 1851	BSE	57
	***Rosenvingea orientalis* (J. Agardh) Børgesen, 1914**	BPG	56, 57
Order Fucales, Family Sargassaceae	*Sargassum liebmannii* J. Agardh 1847	BSE	184
	*Sargassum* sp.	BPG	58
Phylum RHODOPHYTA, Class Bangiophyceae, Order Bangiales, Family Bangiaceae	***Bangia fuscopurpurea* (Dillwyn) Lyngbye, 1819**	BSE	188
	***Pyropia thuretii* (Setchell & E.Y.Dawson) J.E. Sutherland, L.E. Aguilar Rosas & R. Aguilar Rosas, 2011**	BSE	57
Class Compsopogonophyceae, Order Erythropeltidales, Family Erythrotrichiaceaea	***Smithora naiadum* (C.L. Anderson) Hollenberg, 1959 as *Porphyra naiadum***	BSE	188
Class Florideophyceae, Order Acrochaetiaceae, Family Acrochaetiaceae	***Acrochaetium arcuatum* (K.M. Drew) C.K. Tseng, 1945 as *Acrochaetium penetrale***	BSE	57, 188
Order Ceramiales, Family Rhodomelaceae	*Bostrychia* sp.	Jun	185
	***Chondria dangeardii* E.Y. Dawson, 1954 as *Chondria platyclada***	BPG	58
Order Ceramiales, Family Ceramiaceae	*Ceramium avalonae* E.Y. Dawson, 1949	BPG	57
	***Ceramium personatum* Setchell & N.L. Gardner, 1930**	BSE	57, 188
Order Ceramiales, Family Rhodomelaceae	***Neosiphonia beaudettei* (Hollenberg) M.-S. Kim & I.A. Abbott, 2006 as *Polysiphonia beaudettei***	BPG	57
	*Polysiphonia bifurcata* Hollenberg in W.R. Taylor, 1945	PBl	57
Order Gigartinales, Family Dicranemataceae	***Dicranema rosaliae* Setchell & Gardner, 1924**	BSE	57
Order Gracilariales, Family Gracilariaceae	*Gracilaria symmetrica* Dawson, 1949	BPG	58
	*Gracilaria* sp.	BSE	57
	*Gracilariopsis* sp.	BPG	58
Order Hildebrandiales, Family Hildebrandiaceae	*Hildenbrandia rubra* (Sommerfelt) Meneghini, 1841 as *Hildenbrandia prototypus*	BSE	188
Order Rhodymeniales, Family Rhodymeniaceae	***Botryocladia beaudettei* E.Y. Dawson, 1960**	BPG	57
Division MAGNOLIOPHYTA, Class Magnoliopsida, Order Ericales, Family Tetrameristaceae	*Pelliciera rhizophoreae* Triana & Planchon, 1862	BSE	128
	*Pelliciera rhizophoreae* Triana & Planchon, 1862	MPG	32, 128, 208
	*Pelliciera rhizophoreae* Triana & Planchon, 1862	MPN	176
Order Lamiales, Family Acanthaceae	*Avicennia bicolor* Standley, 1923	MJu	32
	*Avicennia bicolor* Standley, 1923	MCa	32
	*Avicennia bicolor* Standley, 1923	MSa	32
	*Avicennia bicolor* Standley, 1923	BSE	128
	*Avicennia bicolor* Standley, 1923	MPG	32, 208
	*Avicennia bicolor* Standley, 1923	MPN	32, 208
	*Avicennia germinans* Linnaeus, 1764	MJu	32
	*Avicennia germinans* Linnaeus, 1764	MCa	32
	*Avicennia germinans* Linnaeus, 1764	MSa	32
	*Avicennia germinans* Linnaeus, 1764	BSE	128
	*Avicennia germinans* Linnaeus, 1764	MPG	32, 128, 208
	*Avicennia germinans* Linnaeus, 1764 as *Avicennia tonduzii* in reference 172	MPN	32, 116, 172, 206
	*Avicennia* spp.	MPN	176
Order Myrtales, Family Combretaceae	*Conocarpus erectus* Linnaeus, 1753	MJu	32
	*Conocarpus erectus* Linnaeus, 1753	MCa	32
	*Conocarpus erectus* Linnaeus, 1753	MSa	32
	*Conocarpus erectus* Linnaeus, 1753 as *Conocarpus erecta* in reference 176	MPN	172, 176, 206
	*Laguncularia racemosa* (L.) Gärtner, 1807	MJu	32
	*Laguncularia racemosa* (L.) Gärtner, 1807	MSa	32
	*Laguncularia racemosa* (L.) Gärtner, 1807	BSE	128
	*Laguncularia racemosa* (L.) Gärtner, 1807	MPG	128, 208
	*Laguncularia racemosa* (L.) Gärtner, 1807	MPN	172, 176, 206
Order Rhizophorales, Family Rhizophoraceae	*Rhizophora mangle* Linnaeus, 1753	MJu	32
	*Rhizophora mangle* Linnaeus, 1753	MCa	32
	*Rhizophora mangle* Linnaeus, 1753	MSa	32
	*Rhizophora mangle* Linnaeus, 1753	BSE	128
	*Rhizophora mangle* Linnaeus, 1753	MPG	32, 128
	*Rhizophora mangle* Linnaeus, 1753	MPN	32, 170, 208
	*Rhizophora racemosa* Meyer, 1818	MJu	32
	*Rhizophora racemosa* Meyer, 1818	MCa	32
	*Rhizophora racemosa* Meyer, 1818	MSa	32
	*Rhizophora racemosa* Meyer, 1818	BSE	128
	*Rhizophora racemosa* Meyer, 1818	MPG	32, 128, 208
	*Rhizophora racemosa* Meyer, 1818	MPN	32, 206, 208
	*Rhizophora* spp.	MPN	176
Phylum FORAMINIFERA, Class Globothalamea, Order Lituolida, Family Discamminidae	***Ammoscalaria compressa* (Cushman & McCulloch, 1939) as *Ammofrondicularia compressa***	PBl	50
Order Lituolida, Family Haplophragmoididae	***Haplophragmoides planissima* Cushman, 1927 as *Haplophragmoides planissimum***	BSE	50
	***Labrospira columbiensis* (Cushman, 1925) as *Haplophragmoides columbiense***	PBl	50
	***Labrospira columbiensis* (Cushman, 1925) as *Haplophragmoides columbiense***	BSE	50
Order Lituolida, Family Lituolidae	*Eratidus foliaceus* (Brady, 1881) as *Ammobaculites foliaceus*	PBl	50
Order Lituolida, Family Reophacidae	***Reophax curtus* Cushman, 1920**	PBl	50
	***Reophax scorpiurus* de Montfort, 1808**	BSE	50
Order Lituolida, Family Nouriidae	*Nouria polymorphinoides* Heron-Allen & Earland, 1914	BSE	50
	*Nouria polymorphinoides* Heron-Allen & Earland, 1914	PBl	50
Order Lituolida, Family Remaneicidae	***Remaneica kellettae* (Thalmann, 1932) as *Trochammina kellettae***	BSE	50
Order Lituolida, Family Trochamminidae	*Portatrochammina pacifica* (Cushman, 1925) as *Trochammina pacifica*	BSE	50
	*Portatrochammina pacifica* (Cushman, 1925) as *Trochammina pacifica*	PBl	50
Order Rotaliida, Family Bolivinitidae	***Bolivina pygmaea* (Brady, 1881)**	PBl	52
	*Loxostomina limbata* (Brady, 1881) as *Loxostoma limbatum*	BSE	52
Order Rotaliida, Family Buliminellidae	***Buliminella elegantissima* (d’Orbigny, 1839)**	PBl	53
Order Rotaliida, Family Elphidiidae	*Elphidium seymourense* McCulloch, 1977 as Elphidium crispum var. subcrispum	PBL	51
Order Rotaliida, Family Heterohelicidae	*Bifarina pacifica* Cushman & McCulloch, 1942	**BSE^a^**	52
Order Textulariida, Family Textulariidae	*Sahulia conica* (d’Orbigny, 1839) as *Textularia conica*	BSE	127
	*Textularia calva* Lalicker, 1940	BSE	127
	*Textularia candeiana* d’Orbigny, 1839	BSE	127
	*Textularia candeiana* d’Orbigny, 1839	PBl	127
	*Textularia corrugata* Herron-Allen & Earland, 1915	PBl	127
	***Textularia foliacea* Heron-Allen & Earland, 1915**	PBl	127
	***Textularia panamensis* Cushman, 1918**	PBl	127
	*Textularia secasensis* Lalicker & McCulloch, 1940	BSE	127
	*Textularia secasensis* Lalicker & McCulloch, 1940	PBl	127
Class Tubothalamea, Order Spirillinida, Family Ammodiscidae	***Glomospira gordialis* (Jones & Parker, 1860)**	BSE	50
Class *incerta sedis*, Order Lagenida, Family Lagenidae	***Lagena amphora* Reuss, 1863**	PBl	54
	*Lagena striata* (d’Orbigny, 1839)	PBl	54
Phylum CNIDARIA, Class Anthozoa, Orden Antipatharia, Family Antipathidae	*Antipathes* sp.	IMu	7
Order Alcyonacea, Family Gorgoniidae	*Eugorgia aurantiaca* (Horn, 1860)	IMu	38
	*Eugorgia daniana* Verrill, 1868	BSE	11
	*Eugorgia rubens* Verrill, 1868	IMu	11, 18
	*Leptogorgia alba* (Duchassaing & Michelotti, 1864)	IBo	11, 15, 38
	*Leptogorgia alba* (Duchassaing & Michelotti, 1864)	BSE	11, 15, 38
	*Leptogorgia alba* (Duchassaing & Michelotti, 1864)	PBl	7
	*Leptogorgia alba* (Duchassaing & Michelotti, 1864)	IMu	7, 11, 15, 38
	*Leptogorgia cofrini* Breedy & Guzman, 2005	**IMu^b^**	14
Order Alcyonacea, Family Gorgoniidae	*Leptogorgia cuspidata* Verrill, 1865	IMu	15
	*Leptogorgia regis* Hickson, 1928	BSE	11, 15
	*Leptogorgia regis* Hickson, 1928	IMu	11, 15
	*Pacifigorgia firma* Breedy & Guzman, 2003	BSE	11
	*Pacifigorgia irene* Bayer, 1951	IMu	7, 11, 12, 13
	*Pacifigorgia rubicunda* Breedy & Guzman, 2003	**IMu^b^**	11, 13
	*Pacifigorgia senta* Breedy & Guzman, 2003	**Cua^a^**	11, 13
	*Pacifigorgia senta* Breedy & Guzman, 2003	PBl	7
	*Pacifigorgia senta* Breedy & Guzman, 2003	**IMu^b^**	7, 11, 13
	*Pacifigorgia stenobrochis* (Valenciennes, 1846)	IMu	11, 13
	*Pacifigorgia tupperi* Breedy & Guzman, 2003	**IMu^a^**	11, 13
Order Alcyonacea, Family Plexauridae	*Muricea austera* Verrill, 1869	PBr	17
	*Muricea plantaginea* (Valenciennes, 1846)	BSE	17
	*Muricea squarrosa* Verrill, 1869	LDa	16
	*Muricea* sp.	ACG	38
Order Actiniaria, Family Nemanthidae	***Nemanthus californicus* Carlgren, 1940**	IMu	72
Order Scleractinia, Family Agariciidae	*Gardineroseris planulata* (Dana, 1846)	IMu	7, 42, 124
	*Pavona clavus* (Dana, 1846)	PSE	38, 39
	*Pavona clavus* (Dana, 1846)	PBl	7
	*Pavona clavus* (Dana, 1846)	IMu	7, 41, 42, 124
	*Pavona clavus* (Dana, 1846)	ACG	39, 42
	*Pavona gigantea* Verrill, 1869	PSE	38, 39, 43
	*Pavona gigantea* Verrill, 1869	PBl	7
	*Pavona gigantea* Verrill, 1869	IMu	7, 42, 124
	*Pavona gigantea* Verrill, 1869	ACG	39, 41, 42
	*Pavona maldivensis* (Gardiner, 1905)	ACG	41
	*Pavona varians* Verrill, 1864	IMu	7, 41
	*Pavona varians* Verrill, 1864	ACG	41
Order Scleractinia, Family Dendrophylliidae	*Cladopsammia eguchii* (Wells, 1982)	IMu	38
	*Tubastraea coccinea* Lesson, 1829 as *Tubastrea tenuilamellosa*	PBl	68
	*Tubastraea coccinea* Lesson, 1829	IDa	154
	*Tubastraea coccinea* Lesson, 1829	BVi	154
	*Tubastraea coccinea* Lesson, 1829 as *Tubastrea coccinea*	PSE	38
	*Tubastraea coccinea* Lesson, 1829 as *Tubastrea coccinea*	IMu	7, 42, 124
	*Tubastraea coccinea* Lesson, 1829 as *Tubastrea coccinea*	ACG	39, 42
Order Scleractinia, Family Pocilloporidae	*Pocillopora damicornis* (Linnaeus, 1758)	PSE	43
	*Pocillopora damicornis* (Linnaeus, 1758)	IMu	7, 41, 42, 124
	*Pocillopora damicornis* (Linnaeus, 1758)	ACG	39, 41
	*Pocillopora elegans* Dana, 1846	BSE	7
	*Pocillopora elegans* Dana, 1846	PBl	7
	*Pocillopora elegans* Dana, 1846	IMu	41, 42, 124
	*Pocillopora elegans* Dana, 1846	ACG	39
Order Scleractinia, Family Pocilloporidae	*Pocillopora eydouxi* Milne Edwards & Haime, 1860	ILC	7, 39
	*Pocillopora eydouxi* Milne Edwards & Haime, 1860	PSE	41, 43
	*Pocillopora eydouxi* Milne Edwards & Haime, 1860	IMu	7, 41, 42, 124
	*Pocillopora eydouxi* Milne Edwards & Haime, 1860	ACG	39
	***Pocillopora inflata* Glynn, 1999**, but see Paz-García et al. 2015	BSE	7
	***Pocillopora inflata* Glynn, 1999**	IMu	42, 86
	*Pocillopora meandrina* Dana, 1846	BSE	38
	*Pocillopora meandrina* Dana, 1846	PSE	41
	*Pocillopora meandrina* Dana, 1846	IMu	7, 41
	*Pocillopora meandrina* Dana, 1846	ACG	39
Order Scleractinia, Family Poritidae	*Porites lobata* Dana, 1846	PBl	7
	*Porites lobata* Dana, 1846	IMu	7, 42, 124
	*Porites lobata* Dana, 1846	ACG	39
	*Porites panamensis* Verrill, 1866	IMu	42, 124
	*Porites panamensis* Verrill, 1866	ACG	39, 41, 42
Order Scleractinia, Family Rhizangiidae	*Oulangia bradleyi* Verrill, 1866	ACG	39
Order Scleractinia, Family Siderastreidae	*Psammocora stellata* (Verrill, 1866)	BSE	7
	*Psammocora stellata* (Verrill, 1866)	PSE	43
	*Psammocora stellata* (Verrill, 1866)	IMu	7, 41, 42
	*Psammocora profundacella* Gardiner, 1898 as *Psammocora superficialis*	BSE	7
Class Hydrozoa, Order Anthoathecata Family Bougainvilliidae	*Garveia gracilis* (Clark, 1876) as *Bimeria gracilis*	BSE	38, 76, 77
Order Leptothecata, Family Aglaopheniidae	*Aglaophenia trifida* Agassiz, 1862 as *Aglaophenia rigida*	PBl	38, 76
Order Leptothecata, Family Campanulariidae	*Clytia fascicularis* Fraser, 1938	PBl	38, 77
	*Clytia gracilis* (Sars, 1850) as *Clytia cylindrica* and as *Gonothyraea gracilis*	BSE	38, 77
	*Clytia universitatis* Torrey, 1904	BSE	38, 77
Order Leptothecata, Family Haleciidae	*Halecium washingtoni* Nutting, 1901	BSE	38, 77
Order Leptothecata, Family Plumularidae	*Plumularia micronema* Fraser, 1938 as *Plummularia micronema*	BSE	38, 76
	*Plumularia micronema* Fraser, 1938 as *Plummularia micronema*	PBl	38, 76
Order Leptothecata, Family Sertulariidae	*Amphisbetia furcata* (Trask, 1857) as *Sertularia furcata*	CSE	76
	*Dynamena crisioides* Lamouroux, 1824 as *Thuiaria tubuliformis*	BSE	38, 76, 77
	*Dynamena crisioides* Lamouroux, 1824 as *Thuiaria tubuliformis*	PBl	38, 77
	*Sertularia distans* (Lamouroux, 1816) as *Sertularia stookeyi*	CSE	76
Orden Siphonophorae, Familia Physaliidae	*Physalia physalis* (Linnaeus, 1758) as *Physalia physalia*	IMu	38
Phylum PLATYHELMINTHES, Class Trematoda Order Plagiorchiida, Family Acanthocolpidae	***Stephanostomum casum* (Linton, 1910)**	Jun	40, 159
Order Plagiorchiida, Family Lecithasteridae	***Trifoliovarium* sp. Yamaguti, 1940 as *Pseudolecithaster***	Cua	159, 178
Order Plagiorchiida, Family Hemiuridae	***Theletrum lamothei* Pérez-Ponce de León, León-Règagnon & Monks, 1998**	**Cua^a^**	159, 178
Order Plagiorchiida, Family Lepocreadiidae	***Hypocreadium myohelicatum* Bravo-Hollis & Manter, 1957**	Jun	159
Class Cestoda, Order Onchoproteocephalidea, Family Onchobothriidae	***Acanthobothrium franus* Marques, Centritto & Stewart, 1997**	**Cua^a^**	138, 178
	***Acanthobothrium inbiorium* Marques, Centritto & Stewart, 1997**	**Jun^a^**	138, 178
Order Trypanorhyncha, Family Pterobothriidae	***Pterobothrioides carvajali* Campbell & Beveridge, 1997**	**Cua^b^**	24, 178
Phylum ACANTHOCEPHALA, Class Palaeacanthocephala, Order Echinorhynchida Family Illiosentidae	*Koronacatha pectinaria* (Van Cleave, 1940)	Jun	149, 178
Phylum MOLLUSCA, Class Gastropoda, Subclass Heterobranchia, Order Unassigned, Family Acteonidae	*Acteon traskii* Stearns, 1897	BSE	191
Infraclass Opisthobranchia, Order Cephalaspidea, Family Acteocinidae	*Acteocina carinata* (Carpenter, 1857)	IDa	191
	*Acteocina carinata* (Carpenter, 1857)	IDa	191
	*Acteocina carinata* (Carpenter, 1857)	BSE	191
	*Acteocina carinata* (Carpenter, 1857)	PBl	191
	*Acteocina carinata* (Carpenter, 1857)	BPG	191
	*Acteocina infrequens* (C. B. Adams, 1852)	BJu	191
	*Acteocina infrequens* (C. B. Adams, 1852)	IDa	191
	*Acteocina infrequens* (C. B. Adams, 1852)	BSE	191
	*Acteocina infrequens* (C. B. Adams, 1852)	BPG	191
	*Acteocina* sp.	BEH	191
Order Cephalaspidea, Family Aglajidae	*Navanax aenigmaticus* (Bergh, 1894)	BJu	191
Order Cephalaspidea, Family Bullidae	*Bulla punctulata* A. Adams in Sowerby, 1850	Jun	134
Order Cephalaspidea, Family Cylichnidae	*Cylichna atahualpa* (Dall, 1908)	BSE	191
	*Cylichnella tabogaensis* (Strong & Hertlein, 1939)	BJu	191
	*Cylichnella tabogaensis* (Strong & Hertlein, 1939)	IDa	191
	*Cylichnella tabogaensis* (Strong & Hertlein, 1939)	BSE	191
	*Cylichnella tabogaensis* (Strong & Hertlein, 1939)	PBl	191
Order Cephalaspidea, Family Haminoeidae	*Atys defuncta* (Baker & Hanna, 1927)	IDa	191
	*Atys defuncta* (Baker & Hanna, 1927)	BSE	191
	*Atys defuncta* (Baker & Hanna, 1927)	IMu	191
	*Atys defuncta* (Baker & Hanna, 1927)	CSE	191
	*Atys defuncta* (Baker & Hanna, 1927)	BPG	191
	*Atys exaratus* (Carpenter, 1857) as *Atys exarata*	BJu	191
	*Atys exaratus* (Carpenter, 1857) as *Atys exarata*	IDA	191
	*Atys exaratus* (Carpenter, 1857) as *Atys exarata*	PBl	191
	*Atys exaratus* (Carpenter, 1857) as *Atys exarata*	IMu	191
Order Cephalaspidea, Family Retusidae	*Retusa paziana* Dall, 1919	IDa	191
	*Retusa paziana* Dall, 1919	BSE	191
	*Retusa* sp.	PNa	191
Order Cephalaspidea, Family Rhizoridae	*Volvulella cylindrica* (Carpenter, 1864)	PJu	191
	*Volvulella cylindrica* (Carpenter, 1864)	BSE	191
	*Volvulella cylindrica* (Carpenter, 1864)	PBl	191
	*Volvulella cylindrica* (Carpenter, 1864)	IMu	191
	*Volvulella cylindrica* (Carpenter, 1864)	BPG	191
	*Volvulella cylindrica* (Carpenter, 1864)	PNa	191
Order Nudibranchia, Family Discodorididae	*Atagema notacristata* Camacho-García & Gosliner 2008	IMu	22
Orden Nudibranchia, Family Fionidae	*Fiona pinnata* (Eschscholtz, 1831)	BSE	130
Orden Nudibranchia, Family Polyceridae	*Limacia janssi* (Bertsch & Ferreira, 1974) as *Laila janssi*	**BSE^a, b^**	8
Order Sacoglossa, Family Plakobranchiadae	*Elysia* sp.	IMu	23
Infraclass Pulmonata, Order Unassigned, Family Siphonariidae	*Siphonaria gigas* Sowerby, 1825	CSE	130
	*Siphonaria gigas* Sowerby, 1825	Jun	185
Infraclass Unassigned, Family Pyramidellidae	*Eulimastoma dotella* (Dall & Bartsch, 1909) as Odostomia (Telloda) subdotella	BSE	114
	*Odostomia costaricensis* Hertlein & Strong, 1951 as Odostomia (Chrysallida) costaricensis	BSE	114
	*Odostomia nicoyana* Hertlein & Strong, 1951 as Odostomia (Menestho) nicoyana	BSE	114
	*Odostomia woodhridgei* Hertlein & Strong, 1951 as Odostomia (Chrysallida) woodbridgei	BSE	114
	Odostomia (Besla) caneloensis Hertlein & Strong, 1951	**BSE^a^**	114
	*Turbonilla amiriana* Hertlein & Strong, 1951 as Turbonilla (Pyrgiscus) amiriana	BSE	114
	*Turbonilla ayamana* Hertlein & Strong, 1951 as Turbonilla (Pyrgiscus) ayamana	BSE	114
	*Turbonilla biolleyi* Hertlein & Strong, 1951 as Turbonilla (Pyrgiscus) biolleyi	BSE	114
	*Turbonilla ekidana* Hertlein & Strong, 1951 as Turbonilla (Pyrgiscus) ekidana	BSE	114
	*Turbonilla guanacastensis* Hertlein & Strong, 1951 as Turbonilla (Pyrgiscus) guanacastensis	BSE	114
	*Turbonilla nicoyana* Hertlein & Strong, 1951 as Turbonilla (Pyrgiscus) nicoyana	BSE	114
	*Turbonilla portoparkerensis* Hertlein & Strong, 1951 as Turbonilla (Ptycheulimella) portoparkensis	BSE	114
	*Turbonilla sulacana* Hertlein & Strong, 1951 as Turbonilla (Pyrgiscus) sulacana	BSE	114
	*Turbonilla templetonis* Hertlein & Strong, 1951 as Turbonilla (Pyrgiscus) templetonis	BSE	114
	*Turbonilla utuana* Hertlein & Strong, 1951 as Turbonilla (Pyrgisculus) utuana	BSE	114
	*Turbonilla zacae* Hertlein & Strong, 1951 as Turbonilla (Pyrgiscus) zacae	BSE	114
Subclass Caenogastropoda, Order Littorinimorpha, Family Cypraeidae	*Pseudozonaria arabicula* (Lamarck, 1810) as Zonaria (Zonaria) arabicula	IMu	26
Order Littorinimorpha, Family Ficidae	*Ficus ventricosa* (G. B. Sowerby I, 1825)	IMu	130
Order Littorinimorpha, Family Littorinidae	*Echinolittorina atrata* (C. B. Adams, 1852) as *Nodilittorina atrata*	PSE	174
	*Echinolittorina fuscolineata* (Reid, 2002) as *Nodilittorina fuscolineata*	IMu	174
	*Echinolittorina modesta* (Philippi, 1846) as *Littorina modesta*	IMu	130
	*Echinolittorina peruviana* (Lamarck, 1822) as *Littoraria zebra*	MCa	32
	*Echinolittorina peruviana* (Lamarck, 1822) as *Littoraria zebra*	MSa	32
	*Echinolittorina peruviana* (Lamarck, 1822) as Littoraria (Littoraria) zebra	BSE	173
	*Echinolittorina peruviana* (Lamarck, 1822) as *Littoraria zebra*	PPG	32, 208
	*Echinolittorina peruviana* (Lamarck, 1822) as *Littoraria zebra*	MPN	32, 208
Order Littorinimorpha, Family Personidae	*Distorsio decussata* (Valenciennes, 1832)	IMu	130
Order Littorinimorpha, Family Rissoinidae	*Zebinella alarconi* (Hertlein & Strong, 1951) as *Rissoina alarconi*	BSE	114
Order Littorinimorpha, Family Strombidae	*Persististrombus granulatus* (Swainson, 1822) as *Strombus granulatus* Swainson, 1822	IMu	130
Order Littorinimorpha, Family Tornidae	*Anticlimax willetti* Hertlein & Strong, 1951 as Anticlimax (Subclimax) willetti	**BSE^a^**	114
	*Aorotrema humboldti* (Hertlein & Strong, 1951) as *Cyclostremiscus humboldti*	BSE	114
	*Teinostoma herbertianum* Hertlein & Strong, 1951 as *Teinostoma herbertiana*	BSE	114
	*Teinostoma zacae* Hertlein & Strong, 1951	BSE	114
Order Neogastropoda, Family Columbellidae	*Anachis fluctuata* (G. B. Sowerby I, 1832) as Anachis (Parvanachis) fluctuata	IMu	130
	*Anachis pardalis* (Hinds, 1843) as Anachis (Parvanachis) carmen	IMu	130
	*Clavistrombina clavulus* (G. B. Sowerby I, 1834)	BSE	125
	*Cosmioconcha rehderi* (Hertlein & Strong, 1951) as *Anachis rehderi*	**BSE^a^**	114
	*Cotonopsis hirundo* (Gaskoin, 1852) as Cotonopsis (Turrina) hirundo	BSE	125
	*Mazatlania fulgurata* (Philippi, 1846) as *Terebra moolenbeeki*	**PNa^a^**	4, 9
	*Sincola dorsata* (G. B. Sowerby I, 1832) as Sincola (Dorsina) dorsata	BSE	125
	*Strombina elegans* (G. B. Sowerby I, 1832) as Strombina (Spiralta) elegans	BSE	125
	*Sincola gibberula* (G. B. Sowerby I, 1832) as Sincola (Dorsina) gibberula	BSE	125
	*Strombina elegans* (G. B. Sowerby I, 1832) as Strombina (Spiralta) elegans	PBl	125
	*Strombina elegans* (G. B. Sowerby I, 1832) as Strombina (Spiralta) elegans	IMu	125
	*Strombina maculosa* (G. B. Sowerby I, 1832) as Strombina (Spiralta) maculosa	BSE	125
	*Strombina maculosa* (G. B. Sowerby I, 1832) as Strombina (Spiralta) maculosa	IMu	125
Order Neogastropoda, Family Columbellidae	*Strombina pulcherrima* (G. B. Sowerby I, 1832) as Strombina (Lirastrombina) pulcherrima	BSE	125
	*Strombina pulcherrima* (G. B. Sowerby I, 1832) as Strombina (Lirastrombina) pulcherrima	IMu	125
	*Strombina recurva* (G. B. Sowerby I, 1832) as Strombina (Recurvina) recurva	BSE	125
	*Strombina recurva* (G. B. Sowerby I, 1832) as Strombina (Recurvina) recurva	PBl	125
	*Strombina recurva* (G. B. Sowerby I, 1832) as Strombina (Recurvina) recurva	BPG	125
	*Strombina solidula* (Reeve, 1859) as Strombina (Lirastrombina) solidula – doubtful record	BSE	125
Order Neogastropoda, Family Conidae	*Conasprella lucida* (W. Wood, 1828) as *Conus lucidus* Wood, 1828	BSE	93
	*Conasprella perplexa* (G. B. Sowerby II, 1857) as *Conus perplexus*	BSE	93
	*Conasprella tornata* (G. B. Sowerby I, 1833) as *Conus tornatus*	BSE	93
	*Conus brunneus* Wood, 1828	BSE	93
	*Conus brunneus* Wood, 1828	IMu	93
	*Conus dalli* Stearns, 1873	BSE	93
	*Conus fergusoni* G. B. Sowerby II, 1873	BSE	93
	*Conus gladiator* Broderip, 1833	BSE	93
	*Conus recurvus* Broderip, 1833	BSE	93
	*Conus vittatus* Hwass in Bruguière, 1792	BSE	93
Order Neogastropoda, Family Fasciolariidae	*Fusinus dupetitthouarsi* (Kiener, 1840)	IMu	130
	*Opeatostoma pseudodon* (Burrow, 1815)	IMu	130
	*Pustulatirus hemphilli* (Hertlein & Strong, 1951) as *Latirus hemphilli*	**BSE^a^**	114
	*Pustulatirus mediamericanus* (Hertlein & Strong, 1951) as *Latirus mediamericanus*	BSE	114
Order Neogastropoda, Family Marginellidae	*Dentimargo zetetes* Roth, 1978	**BSE^a^**	181
	*Prunum aletes* Roth, 1978 as Prunum (microspira) aletes	BSE	181
	*Prunum aletes* Roth, 1978 as Prunum (microspira) aletes	CSE	181
	*Prunum aletes* Roth, 1978 as Prunum (microspira) aletes	**IMu^a^**	181
	*Prunum lizanoi* Magaña, Espinosa & Ortea, 2003	**BJu^a^**	133, 199
Order Neogastropoda, Family Muricidae	*Acanthina* sp.	CSE	130
	*Murexsul zeteki* (Hertlein & Strong, 1951) as *Muricopsis zeteki*	BSE	114
	*Plicopurpura columellaris* (Lamarck, 1816) as *Purpura pansa*	CSE	130
	*Vasula melones* (Duclos, 1832)	BEH	185
Order Neogastropoda, Family Pseudomelatomidae	*Crassispira xanti* Hertlein & Strong, 1951	BSE	114
Order Unassigned, Family Cerithiopsidae	*Cerithiopsis guanacastensis* Hertlein & Strong, 1951	**BSE^a^**	114
	*Seila kanoni* (de Folin, 1867)	BSE	69
	*Seila montereyensis* Bartsch, 1907	CSE	69
Order Unassigned, Family Potamididae	*Cerithideopsis californica* (Haldeman, 1840) as *Cerithidea valida*	MCa	32
Order Unassigned, Family Potamididae	*Cerithideopsis californica* (Haldeman, 1840) as *Cerithidea valida*	MSa	32
	*Cerithideopsis californica* (Haldeman, 1840) as *Cerithidea valida*	PPG	32, 203
	*Cerithideopsis californica* (Haldeman, 1840) as *Cerithidea valida*	MPN	30, 208
Subclass Neritimorpha, Order Cycloneritimorpha, Family Neritidae	*Nerita costata* Gmelin, 1791 as *Nerita scabricosta*	BSE	128
	*Nerita costata* Gmelin, 1791 as *Nerita scabricosta*	IMu	130
Subclass Vetigastropoda, Order Unassigned, Family Scissurellidae	*Scissurella kaiserae* Geiger, 2006	CSE	85
Class Bivalvia, Subclass Heterodonta, Order Cardiida, Family Cardiidae	*Americardia biangulata* (Broderip & G. B. Sowerby I, 1829) as *Cardium biangulatum*	BSE	109
	*Laevicardium substriatum* (Conrad, 1837) as *Cardium alenense*	BSE	109
	*Lophocardium annettae* (Dall, 1889) as *Cardium annettae*	BSE	109
	*Microcardium pazianum* (Dall, 1916) as *Cardium pazianum*	BSE	109
	*Trachycardium consors* (G. B. Sowerby I, 1833) as *Cardium consors*	BSE	109
	*Trachycardium procerum* (G. B. Sowerby I, 1833) as *Cardium procerum*	BSE	109
	*Trigoniocardia granifera* (Broderip & G. B. Sowerby I, 1829) as *Cardium graniferum*	BSE	109
Order Cardiida, Family Psammobilidae	*Heterodonax bimaculatus* (Linnaeus, 1758) as *Heterodonax bimaculata*	BSE	113
Order Cardiida, Family Semelidae	*Cumingia lamellosa* G. B. Sowerby I, 1833	BSE	112
	*Semele jovis* (Reeve, 1853)	BSE	112
	*Semele pallida* (G. B. Sowerby I, 1833) as *Semele simplicissima*	BSE	112
	*Semele verrucosa* Mörch, 1860 as *Semele pacifica*	BSE	112
Order Cardiida, Family Solecurtidae	*Tagelus affinis* (C. B. Adams, 1852) as Tagelus (Tagelus) affinis	BSE	113
	*Tagelus politus* (Carpenter, 1857) as Tagelus (Mesopleura) politus	BSE	113
Order Cardiida, Family Tellinidae	*Cymatoica undulata* (Hanley, 1844) as Macoma (Cymatoica) undulata	BSE	111
	*Macoma panamensis* Dall, 1900 as Macoma (Psammacoma) panamensis	BSE	111
	*Tellina amianta* Dall, 1900 as Tellina (Moerella) amianta	BSE	111
	*Tellina inaequistriata* Donovan, 1802 as Tellina (Eurytellina) inaequistriata	BSE	111
	*Tellina martinicensis* d’Orbigny, 1853 as Tellina (Merisca) proclivis	BSE	111
	*Tellina pristiphora* Dall, 1900 as Tellina (Phyllodina) pristiphora	BSE	111
	*Tellina prora* Hanley, 1844 as Tellina (Eurytellina) prora	BSE	111
	*Tellina rubescens* Hanley, 1844 as Tellina (Eurytellina) rubescens	BSE	111
	*Tellina tabogensis* Salisbury, 1934 as Tellina (Moerella) recurvata	BSE	111
Order Carditida, Family Carditidae	*Carditamera affinis* (G. B. Sowerby I, 1833) as Cardita (Carditamera) affinis	BSE	108
	*Carditamera radiata* (G. B. Sowerby I, 1833) as Cardita (Carditamera) radiate	BSE	108
	*Cardites laticostatus* (G. B. Sowerby I, 1833) as *Cardita tricolor*	BSE	108
	*Strophocardia megastropha* (J.E. Gray, 1825) as *Cardita megastropha*	BSE	108
Order Carditida, Family Condylocardiidae	*Condylocardia sparsa* Coan, 2003	CSE	29
Order Carditida, Family Crassatellidae	*Crassinella pacifica* (C. B. Adams, 1852)	BSE	108
	*Eucrassatella antillarum* (Reeve, 1842) as Crassatellites (Hybolophus) digueti	BSE	108
	*Eucrassatella gibbosa* (G. B. Sowerby I, 1832) as Crassatellites (Hybolophus) gibbosus	BSE	108
Order Lucinida, Family Lucinidae	*Codakia distinguenda* (Tryon, 1872)	BSE	108
	*Ctena mexicana* (Dall, 1901)	BSE	108
	*Liralucina approximata* (Dall, 1901) as Lucina (Parvillucina) approximata	BSE	108
	*Radiolucina cancellaris* (Philippi, 1846) as Lucina (Bellucina) cancellaris	BSE	108
Order Myida, Family Corbulidae	*Caryocorbula biradiata* (G. B. Sowerby I, 1833) as Aloidis (Caryocorbula) biradiata	BSE	113
	*Caryocorbula marmorata* (Hinds, 1843) as Aloidis (Caryocorbula) marmorata	BSE	113
	Caryocorbula nasuta (G. B. Sowerby I, 1833) as Aloidis (Caryocorbula) nasuta	BSE	113
Order Myida, Family Pholadidae	*Jouannetia pectinata* (Conrad, 1849) as Jouannetia (Triomphalia) pectinata	BSE	113
Order Venerida, Family Veneridae	*Agriopoma catharium* (Dall, 1902) as Pitar (Pitarella) mexicanus	BSE	110
	*Anomalocardia subimbricata* (Sowerby, 1835)	BSE	110
	*Chione compta* (Broderip, 1835) as Chione (Chione) compta	BSE	110
	*Cyclinella subquadrata* (Hanley, 1844)	BSE	110
	*Dosinia dunkeri* (Philippi, 1844) as Dosinia (Dosinidia) dunkeri	BSE	110
	*Dosinia ponderosa* (Gray, 1838) as Dosinia (Dosinidia) ponderosa	BSE	110
	*Gouldia californica* Dall, 1917	BSE	110
	*Iliochione subrugosa* (W. Wood, 1828) as *Anomalocardia subrugosa*	BSE	110
	*Leukoma asperrima* (G. B. Sowerby I, 1835) as Chione (Nioche) asperrima	BSE	110
	*Lirophora mariae* (d’Orbigny, 1846) as Chione (Lirophora) mariae	BSE	110
	*Megapitaria aurantiaca* (G. B. Sowerby I, 1831)	BSE	110
	*Megapitaria squalida* (G. B. Sowerby I, 1835)	BSE	110
	*Periglypta multicostata* (G. B. Sowerby I, 1835) as Antigona (Periglypta) multicostata	BSE	110
	*Pitar consanguineus* (C. B. Adams, 1852) as Pitar (Pitar) consanguineous	BSE	110
	*Pitar unicolor* (Sowerby, 1835)	IMu	130
	*Protothaca grata* (Say, 1830)	MCa	32
Order Venerida, Family Veneridae	*Protothaca grata* (Say, 1830)	MSa	32
	*Protothaca grata* (Say, 1830) as Protothaca (Callithaca) grata	BSE	110
	*Protothaca grata* (Say, 1830)	MPG	32
	*Protothaca grata* (Say, 1830)	MPN	32
Order Unassigned, Family Chamidae	*Arcinella californica* (Dall, 1903) as *Echinochama californica*	BSE	108
	*Chama echinata* Broderip, 1835	Jun	185
Order Unassigned, Family Galeommatidae	*Bellascintilla parmaleeana* Coney, 1990	PNa	31
Order Unassigned, Family Ungulinidae	*Diplodonta semirugosa* Dall, 1899 as *Taras semirugosus*	BSE	109
	*Diplodonta subquadrata* Carpenter, 1856 as *Taras subquadratus*	BSE	109
Subclass Protobranchia, Order Nuculanida, Family Nuculanidae	*Saccella elenensis* (G. B. Sowerby I, 1833) as Nuculana (Saccella) elenensis	BSE	105
	*Saccella impar* (Pilsbry & Lowe, 1932) as Nuculana (Saccella) impar	BSE	105
	*Saccella laeviradius* (Pilsbry & Lowe, 1932) as Nuculana (Saccella) laeviradius	BSE	105
Subclass Pteriomorphia, Order Arcida, Family Arcidae	*Acar gradata* (Broderip & Sowerby, 1829) as Acar (Arca) gradata	BSE	105
	*Acar gradata* (Broderip & Sowerby, 1829)	PBl	180
	*Anadara biangulata* (G. B. Sowerby I, 1833) as Acar (Anadara) biangulata	BSE	106
	*Anadara nux* (G. B. Sowerby I, 1833) as Arca (Cunearca) nux	BSE	106, 180
	*Anadara perlabiata* (Grant & Gale, 1931) as Arca (Cunearca) perlabiata	BSE	106
	*Anadara reinharti* (Lowe, 1935) as Arca (Anadara) reinharti	BSE	106
	*Anadara reinharti* (Lowe, 1935) as Arca (Scapharca) reinharti	BSE	180
	*Anadara tuberculosa* (G. B. Sowerby I, 1833)	MCa	32
	*Anadara tuberculosa* (G. B. Sowerby I, 1833)	MSa	32
	*Anadara tuberculosa* (G. B. Sowerby I, 1833)	PPG	32, 208
	*Anadara tuberculosa* (G. B. Sowerby I, 1833)	MPN	32
	*Arca mutabilis* (G. B. Sowerby I, 1833) as Arca (Arca) mutabilis	BSE	106, 180
	*Arca mutabilis* (G. B. Sowerby I, 1833) as Arca (Arca) mutabilis	PBl	180
	*Barbatia illota* (G. B. Sowerby I, 1833) Barbatia (Fugleria) illota	PBl	180
	*Barbatia reeveana* (d’Orbigny, 1846) as Barbatia (Cucullaearca) reeveana	PBl	180
	*Calloarca alternata* (G. B. Sowerby I, 1833) as Arca (Calloarca) alternata	BSE	106
	*Larkinia grandis* (Broderip & G. B. Sowerby I, 1829) as *Grandiarca grandis*	MCa	32
	*Larkinia grandis* (Broderip & G. B. Sowerby I, 1829) as Arca (Lakinia) grandis	BSE	106
	*Larkinia grandis* (Broderip & G. B. Sowerby I, 1829) as *Grandiarca grandis*	MPG	32, 208
	*Larkinia grandis* (Broderip & G. B. Sowerby I, 1829) as *Grandiarca grandis*	MPN	32
Subclass Pteriomorphia, Order Arcida, Family Arcidae	*Larkinia multicostata* (G. B. Sowerby I, 1833) as *Anadara multicostata*	MCa	32
	*Larkinia multicostata* (G. B. Sowerby I, 1833) as *Anadara multicostata*	MSa	32
	*Larkinia multicostata* (G. B. Sowerby I, 1833) as Arca (Larkinia) multicostata	BSE	106
	*Larkinia multicostata* (G. B. Sowerby I, 1833) as *Anadara multicostata*	MPG	32, 204
	*Larkinia multicostata* (G. B. Sowerby I, 1833) as *Anadara multicostata*	MPN	32
Order Arcida, Family Glycymerididae	*Tucetona strigilata* (G. B. Sowerby I, 1833) as Glycymeris (Tuceta) tessellata strigilata and as Glycymeris (Tuceta) tessellata	BSE	106
Order Arcida, Family Noetiidae	*Arcopsis solida* (G. B. Sowerby I, 1833) as Arca (Arcopsis) solida	BSE	106
	*Arcopsis solida* (G. B. Sowerby I, 1833)	PBl	180
Order Arcida, Family Philobryidae	*Philobrya setosa* (Carpenter, 1864)	CSE	30
Order Limida, Family Limidae	*Limaria orbignyi* (Lamy, 1930) as Lima (Limaria) orbignyi	BSE	107
Order Mytilida, Family Mytilidae	*Amygdalum americanum* Soot-Ryen, 1955	PBl	186
	*Brachidontes adamsianus* (Dunker, 1856) as *Hormomya adamsiana*	BSE	186
	*Brachidontes puntarenensis* (Pilsbry & Lowe, 1932)	BSE	186
	*Brachidontes* sp.	Jun	185
	*Crenella divaricata* (Orbigny, 1853)	BSE	107, 186
	*Leiosolenus aristatus* (Dillwyn, 1817) as Lithophaga (Myoforceps) aristata	BSE	107, 186
	*Leiosolenus aristatus* (Dillwyn, 1817) as Lithophaga (Myoforceps) aristata	PBl	186
	*Leiosolenus attenuatus* (Deshayes, 1836) as Lithophaga (Labis) attenuata	BSE	107, 186
	*Leiosolenus attenuatus* (Deshayes, 1836) as Lithophaga (Labis) attenuata	PBl	186
	*Leiosolenus plumula* (Hanley, 1843) as Lithophaga (Diberus) plumula	BSE	107, 186
	*Leiosolenus plumula* (Hanley, 1843) as Lithophaga (Diberus) plumula	PBl	186
	*Modiolus capax* (Conrad, 1837) as Volsella (Volsella) capax in reference 107	BSE	107, 186
	*Modiolus capax* (Conrad, 1837)	PBl	186
	*Mytilus* sp.	MCa	32
	*Mytilus* sp.	MSa	32
	*Mytilus* sp.	MPG	32
	*Mytilus* sp.	MPN	32
	*Septifer zeteki* Hertlein & Strong, 1946	BSE	186
Order Ostreida, Family Ostreidae	*Crassostrea corteziensis* (Hertlein, 1951)	MPG	208
	*Saccostrea palmula* (Carpenter, 1857)	Jun	185
	*Saccostrea palmula* (Carpenter, 1857)	BSE	107
Order Ostreida, Family Pinnidae	*Pinna rugosa* G. B. Sowerby I, 1835	BSE	106
Order Ostreida, Family Pteriidae	*Pinctada mazatlanica* (Hanley, 1856)	BSE	106
	*Pinctada mazatlanica* (Hanley, 1856)	IMu	7
	*Pteria sterna* (Gould, 1851)	IMu	7
Order Pectinida, Family Anomiidae	*Placunanomia cumingii* Broderip, 1832	BSE	107
Order Pectinida, Family Pectinidae	*Argopecten irradians concentricus* (Say, 1822) as Pecten (Plagioctenium) circularis	BSE	107
	*Euvola vogdesi* (Arnold, 1906) as Pecten (Pecten) vogdesi	BSE	107
	*Leopecten sericeus* (Hinds, 1845) as Pecten (Pecten) sericeus	BSE	107
	*Leptopecten biolleyi* (Hertlein & Strong, 1946) as Pecten (Leptopecten) velero biolleyi	**BSE^a^**	107
	*Nodipecten subnodosus* (G. B. Sowerby I, 1835) as Pecten (Lyropecten) subnodosus	BSE	104, 107
Order Pectinida, Family Propeamussiidae	*Cyclopecten pernomus* (Hertlein, 1935)	BSE	107
Order Pectinida, Family Spondylidae	*Spondylus* sp.	IMu	7
Phylum SIPUNCULA, Class Phascolosomatidea, Order Phascolosomatida, Family Phascolosomatidae	*Antillesoma antillarum* (Grube & Oersted 1858)	Jun	185
	*Antillesoma antillarum* (Grube & Oersted 1858)	IMu	55, 59
	Phascolosoma (Phascolosoma) perlucens Baird, 1868	IMu	59
	*Phascolosoma* sp.	Jun	185
	Sipunculus (Sipunculus) nudus Linnaeus, 1766	Jun	185
Phylum ANNELIDA, Class Polychaeta, Subclass Errantia, Order Amphinomida, Family Amphinomidae	*Eurythoe complanata* (Pallas, 1776) as *Eurythoe complanata* in reference 95	BSE	60, 95, 189
	*Eurythoe complanata* (Pallas, 1776) as *Eurythoe complanata* in reference 95	PBl	60, 95
	***Hermodice carunculata* (Pallas, 1766)** doubtful record	BSE	60, 189
	*Notopygos ornata* Grube, 1856	BSE	189
	*Notopygos ornata* Grube, 1856	PBl	60, 95
Order Eunicida, Family Eunicidae	***Nicidion mutilata* (Webster, 1884) as *Eunice mutilata***	BSE	60, 96
	***Palola siciliensis* (Grube, 1840)**	BSE	60, 96
Order Eunicida, Family Lumbrineridae	One species	PJu	185
	*Scoletoma tetraura* (Schmarda, 1861) as *Lumbrineris tetraura*	BSE	60, 96
Order Eunicida, Family Oenonidae	***Oenone fulgida* (Savigny in Lamarck, 1818) as *Aglaurides fulgida* in reference 96**	BSE	60, 96
	***Oenone fulgida* (Savigny in Lamarck, 1818) as *Aglaurides fulgida* in reference 96**	PBl	60, 96
Order Eunicida, Family Onuphidae	*Diopatra tridentata* Hartman, 1944	BSE	60, 96
	***Hyalinoecia juvenalis* Moore, 1911**	BSE	60, 96,189
	***Hyalinoecia juvenalis* Moore, 1911**	PBl	60
Order Phyllodocida, Family Glyceridae	One species	PJu	185
	*Glycera tesselata* Grube, 1840	PBl	95
Order Phyllodocida, Family Iphionidae	***Iphione ovata* Kinberg, 1855**	BSE	60, 94
Order Phyllodocida, Family Nereididae	One species	PJu	185
Order Phyllodocida, Family Polynoidae	*Lepidasthenia varius* Treadwell, 1917 as *Lepidasthenia picta* in reference 189	BSE	60, 189
Order Phyllodocida, Family Sigalionidae	***Pelogenia antipoda* Schmarda, 1861 as *Psammolyce antipoda* (Schmarda) *anoculata***	PBl	60, 94
	***Sigalion lewisii* Berkeley & Berkeley, 1939 as *Thalenessa lewisii***	BSE	94
	*Sthenelais fusca* Johnson 1897 as *Stenelais variabilis colorata*	BSE	94
Subclass Sedentaria, Order Spionida, Family Magelonidae	*Magelona* sp.	PJu	185
Order Terebellida, Family Terebellidae	One species	PJu	185
	***Lanicola guillermoi* Capa & Hutchings, 2006**	BEH	185
	***Terebella gorgonae* Monro, 1933**	BSE	60, 189
Order Unassigned, Family Chaetopteridae	One species	PJu	185
Order Unassigned, Family Opheliidae	*Armandia maculata* (Webster, 1884) as *Ammotrypane bermudiensis*	BSE	60, 189
Phylum NEMERTEA	One species	PJu	185
Phylum ARTHROPODA, Subphylum Crustacea Class Malacostraca, Order Amphipoda, Family Ampeliscidae	***Ampelisca brevisimulata* Barnard, 1954**	BSE	5
	***Ampelisca cristata* Holmes, 1908**	BSE	5
	*Ampelisca hancocki* Barnard, 1954	**BSE^a^**	5
	***Ampelisca lobata* Holmes, 1908**	BSE	5
	***Ampelisca milleri* Barnard, 1954**	BSE	5
	***Ampelisca milleri* Barnard, 1954**	PBl	5
	*Ampelisca pugetica* Stimpson, 1864 as Ampelisca pugetica forma macrodentata	BSE	5
	***Ampelisca romigi* Barnard, 1954 as *Ampelisca isocornea***	BSE	5
	*Ampelisca schellenbergi* Shoemaker, 1933	PBl	5
	***Ampelisca venetiensis* Shoemaker, 1916**	BSE	5
Order Amphipoda, Family Aoridae	*Paramicrodeutopus schmitti* (Shoemaker, 1942) as *Microdeutopus schmitti*	PBl	152
Order Amphipoda, Family Neomegamphopidae	*Neomegamphopus roosevelti* Shoemaker, 1942	PBl	152
Order Amphipoda, Family Phoxocephalidae	***Microphoxus minimus* Barnard, 1954**	**PBl^a^**	5, 6
Order Amphipoda, Family Unciolidae	***Acuminodeutopus periculosus* Barnard, 1969 as *Acuminodetopus heteruropus***	PBl	152
Order Amphipoda	Several unidentified species	IMu	75
Order Cumacea, Family Bodotriidae	***Cyclaspis vargasae* Petrescu & Heard, 2004**	**IMu^a^**	161, 162
Order Decapoda, Family Albuneidae	*Lepidopa mearnsi* Benedict, 1904	PNa	197
Order Decapoda, Family Alpheidae	*Alpheus aequus* Kim & Abele, 1988	**PBl^a^**	126, 204
	*Alpheus cylindricus* Kingsley, 1878	BSE	126
	*Alpheus galapagensis* Sivertsen, 1933 as *Alpheus canalis* Kim & Abele, 1988	BSE	126
	*Alpheus galapagensis* Sivertsen, 1933 as *Alpheus canalis* Kim & Abele, 1988	PBl	126
	*Alpheus hebes* Kim & Abele, 1988	BSE	126
	*Alpheus hebes* Kim & Abele, 1988	PBl	126
	*Alpheus longinquus* Kim & Abele, 1988	BSE	126
	*Alpheus longinquus* Kim & Abele, 1988	PBl	126
	*Alpheus normanni* Kingsley, 1878	BSE	126
	*Alpheus panamensis* Kingsley, 1878	CSE	196
	*Alpheus paracrinitus* Miers, 1881	PBl	126
	*Alpheus rostratus* Kim & Abele, 1988	BSE	126, 204
	*Alpheus sulcatus* Kingsley, 1870	CSE	196
	*Alpheus umbo* Kim & Abele, 1988	BSE	126
	*Alpheus* sp.	BEH	185
	*Pomagnathus corallinus* Chace, 1962	BSE	196
	*Pomagnathus corallinus* Chace, 1962	PBl	202
	*Synalpheus digueti* Coutière, 1909	BSE	202
Order Decapoda, Family Axiidae	*Axiopsis serratifrons* (H. Milne-Edwrads, 1873)	IMu	196
Order Decapoda, Family Calappidae	*Calappa convexa* Saussure, 1853	BSE	81
	*Calappula saussurei* (Rathbun, 1898) as *Calappa saussurei*	BSE	198
	*Cryptosoma bairdii* (Stimpson, 1860) as *Cycloes bairdii*	BSE	82
	*Cryptosoma bairdii* (Stimpson, 1860) as *Cycloes bairdii*	IMu	82
	*Platymera gaudichaudii* H. Milne-Edwards, 1837 as *Mursia gaudichaudii*	CSE	129
	*Platymera gaudichaudii* H. Milne Edwards, 1837	IMu	198
Order Decapoda, Family Callianideidae	*Callianidea mariamartae* Hernáez & Vargas, 2013	**IMu^a^**	102
	*Paracallianidea laevicauda* (Gill, 1859) as *Callianidea laevicauda*	PBl	196
Order Decapoda, Family Coenobitidae	*Coenobita compressus* H. Milne Edwards, 1837	IMu	197
Order Decapoda, Family Dairidae	*Daira americana* Stimpson, 1860	BSE	47
Order Decapoda, Family Daldorfiidae	*Daldorfia trigona* (A. Milne-Edwards, 1869) as *Daldorfia garthi*	BSE	47
	*Daldorfia trigona* (A. Milne-Edwards, 1869) as *Daldorfia garthi*	PBl	78
Order Decapoda, Family Diogenidae	*Aniculus elegans* Stimpson, 1858	IMu	197
	*Trizopagurus magnificus* (Bouvier, 1898)	IMu	197
Order Decapoda, Family Domeciidae	*Cherusius triunguiculatus* (Borradaile, 1902) as *Maldivia galapagensis*	BSE	83
Order Decapoda, Family Dromiidae	*Hypoconcha panamensis* Smith, 1869	BSE	82
	*Moreiradromia sarraburei* (Rathbun, 1910)	BSE	198
Order Decapoda, Family Dynomenidae	*Hirsutodynomene ursula* (Stimpson, 1860) as *Dynomene ursula* Stimpson, 1860	IMu	151
Order Decapoda, Family Epialtidae	*Herbstia pubescens* Stimpson, 1871	BSE	80
	*Herbstia pubescens* Stimpson, 1871	PBl	79
	*Macrocoeloma maccullochae* Garth, 1940	BSE	79, 80
	*Macrocoeloma maccullochae* Garth, 1940	PBl	78
	*Microlissa aurivilliusi* (Rathbun, 1898) as *Lissa aurivilliusi*	BSE	80
	*Microlissa aurivilliusi* (Rathbun, 1898) as *Lissa aurivilliusi*	PBl	79
	*Pelia tumida* (Lockington, 1877)	PBl	79
	*Stenocionops ovatus* (Bell, 1835) as *Stenocionops ovata*	IMu	198
Order Decapoda, Family Eriphiidae	*Eriphia squamata* Stimpson, 1859	BSE	47
	*Eriphides hispida* (Stimpson, 1860)	BSE	47
Order Decapoda, Family Ethusidae	*Ethusa lata* Rathbun, 1893	BSE	81
	*Ethusa lata* Rathbun, 1893	IMu	198
	*Ethusa panamensis* Finnegan, 1931 as *Ethusa mascarpone panamensis*	IMu	82
Order Decapoda, Family Gecarcinidae	*Johngarthia planata* (Stimpson, 1860)	IMu	160
Order Decapoda, Family Grapsidae	*Geograpsus lividus* (Milne Edwards, 1837)	BSE	47
	*Grapsus grapsus* (Linnaeus, 1758)	BSE	47
	*Grapsus grapsus* (Linnaeus, 1758)	PPG	47
	*Pachygrapsus transversus* (Gibbes, 1850)	BSE	47
Order Decapoda, Family Hippidae	*Emerita rathbunae* Schmitt, 1935	Jun	197
Order Decapoda, Family Hippolytidae	*Hippolyte williamsi* Schmitt, 1924	IMu	201
	*Lysmata argentopunctata* Wicksten, 2000	IMu	201, 204
	*Trachycaris restricta* (Milne-Edwards, 1878)	BSE	196
Order Decapoda, Family Inachidae	*Ericerodes angulatus* (Finnegan, 1931) as *Podochela angulata*	BSE	79
	*Ericerodes angulatus* (Finnegan, 1931) as *Podochela angulata*	BSE	80
	*Ericerodes veleronis* (Garth, 1948) as *Podochela veleronis*	PBl	79
	*Eucinetops panamensis* Rathbun, 1923	BSE	47
	*Eucinetops panamensis* Rathbun, 1923	PBl	79
	*Podochela ziesenhennei* Garth, 1940	PBl	79
Order Decapoda, Family Inachoididae	*Collodes tenuirostris* Rathbun, 1894	IMu	198
	*Euprognatha bifida* Rathbun, 1893	BSE	80
	*Inachoides laevis* Stimpson, 1860	BSE	79
	*Inachoides laevis* Stimpson, 1860	BSE	80
	*Paradasygyius depressus* (Bell, 1835)	BSE	79
	*Pyromaia tuberculata* (Lockington, 1877)	IMu	198
	*Stenorhynchus debilis* (Smith, 1871)	BSE	79
	*Stenorhynchus debilis* (Smith, 1871)	PBl	79
	*Stenorhynchus debilis* (Smith, 1871)	BSE	80
Order Decapoda, Family Leucosiidae	*Ebalia magdalenensis* Rathbun, 1933	BSE	82
	*Leucosilia jurinii* (Saussure, 1853)	BSE	82
	*Lithadia cumingii* Bell, 1855	BSE	82
	*Persephona subovata* (Rathbun, 1894) as *Iliacantha hancocki*	BSE	82
	*Randallia agaricias* Rathbun, 1898	BSE	82
Order Decapoda, Family Majidae	*Maiopsis panamensis* Faxon, 1893	IMu	198
Order Decapoda, Family Mithracidae	*Ala cornuta* (Stimpson, 1860) as *Anaptychus cornutus*	BSE	47
	*Ala cornuta* (Stimpson, 1860)	BSE	79, 80
	*Ala cornuta* (Stimpson, 1860)	PBl	79
	*Hemus finneganae* Garth, 1958	BSE	80
	*Microphrys branchialis* Rathbun, 1898	BSE	79
	*Microphrys platysoma* (Stimpson, 1860)	BSE	47
	*Mithraculus denticulatus* (Bell, 1835) as *Mithrax denticulatus*	BSE	47
	*Mithraculus denticulatus* (Bell, 1835) as *Mithrax denticulatus*	PBl	79
	*Mithrax tuberculatus* Stimpson, 1860	PBl	79
	*Petramithrax pygmaeus* Bell, (1836) as *Mithrax pygmaeus*	BSE	47, 80
	*Pitho picteti* (Saussure, 1853)	PBl	79
	*Pitho picteti* (Saussure, 1853)	BSE	80
	*Pitho quinquedentata* Bell, 1835	BSE	80
	*Pitho sexdentata* Bell, 1835	BSE	47
	*Teleophrys cristulipes* Stimpson, 1860	BSE	47, 79
	*Teleophrys cristulipes* Stimpson, 1860	PBl	79
	*Thoe erosa* Bell, 1835 as *Thoe sulcata panamensis*	BSE	47, 79
	*Thoe erosa* Bell, 1835 as *Thoe sulcata panamensis*	PBl	79
Order Decapoda, Family Munididae	*Pleuroncodes planipes* Stimpson, 1860	CSE	129
Orden Decapoda, Family Ocypodidae	*Ocypode gaudichaudii* Milne Edwards & Lucas, 1843	BPG	44, 46
	Uca (Leptuca) deichmanni Rathbun, 1935 as *Uca deichmanni*	BSE	45
	Uca (Leptuca) latimanus (Rathbun, 1894) as *Uca latimanus*	BSE	45
	Uca (Leptuca) panamensis (Stimpson, 1859) as *Uca panamensis*	BSE	45
	Uca (Leptuca) stenodactylus (H. Milne Edwards & Lucas, 1843) as *Uca stenodactyla*	BSE	45
	Uca (Leptuca) terpsichores Crane, 1941 as *Uca terpsichores*	BSE	45
	Uca (Minuca) brevifrons (Stimpson, 1860) as *Uca brevifrons*	BSE	45
	*Uca* sp.	MCa	32
	*Uca* sp.	MSa	32
	*Uca* sp.	MPG	32, 208
	*Uca* sp.	MPN	32
Order Decapoda, Family Oziidae	*Epixanthus tenuidactylos* (Lockington, 1877) as *Ozius tenuidactylus*	BSE	47
	*Ozius verreauxii* Saussure, 1853	BSE	47
Order Decapoda, Family Paguridae	*Pagurus vetaultae* Harvey & McLaughlin, 1991	**BSE^b^**	97
	*Pagurus virgulatus* Haig & Harvey, 1991	**BSE^a^**	92, 197
Order Decapoda, Family Palaemonidae	*Ancylomenes lucasi* (Chace, 1937) as *Periclimenes lucasi*	PBl	118
	*Brachycarpus biunguiculatus* (Lucas, 1846)	BSE	119
	*Brachycarpus biunguiculatus* (Lucas, 1846)	PBl	119
	*Brachycarpus biunguiculatus* (Lucas, 1846)	IMu	196
	*Fennera chacei* Holthuis, 1951	BSE	118
	*Periclimenes infraspinis* (Rathbun, 1902) as *Periclimenaeus infraspinis*	BSE	118, 202, 204
	***Periclimenes murcielagensis* Vargas, 2000**	**IMu^a^**	194, 204
	*Pontonia margarita* Smith, 1869	BSE	118
	*Pontonia margarita* Smith, 1869	PBl	118
	*Pontonia margarita* Smith, 1869	IMu	196
	*Pontonia simplex* Holthuis, 1951	BSE	196
	*Waldola schmitti* Holthuis, 1951	IMu	196
Order Decapoda, Family Panopeidae	*Eurypanopeus planus* (Smith, 1869)	BSE	47
	*Eurypanopeus transversus* (Stimpson, 1860)	BSE	47
	*Hexapanopeus costaricensis* Garth, 1940	**BSE^a^**	78, 81
	*Hexapanopeus orcutti* Rathbun, 1930	BSE	81
	*Hexapanopeus sinaloensis* Rathbun, 1930	BSE	81
	*Malacoplax californiensis* (Lockington, 1877) as *Speocarcinus californiensis*	BSE	81
Order Decapoda, Family parthenopidae	*Celatopesia hassleri* (Rathbun, 1925) as *Cryptopodia hassleri*	BSE	79, 80
	*Celatopesia hassleri* (Rathbun, 1925) as *Cryptopodia hassleri*	PBl	79
	*Heterocrypta macrobrachia* Stimpson, 1871	BSE	79
	*Hypolambrus hyponcus* (Stimpson, 1871) as Pathenope (Pathenope) hyponca	PBl	79
	*Mesorhoea bellii* (A. Milne-Edwards, 1878)	BSE	198
	*Solenolambrus arcuatus* Stimpson, 1871	BSE	79, 80
	*Solenolambrus arcuatus* Stimpson, 1871	PBl	79
	*Spinolambrus exilipes* (Rathbun, 1893) as *Parthenope exilipes*	IMu	198
Order Decapoda, Family Pasiphaeidae	Leptochela (Leptochela) serratorbita Spence Bate, 1888 as *Leptochela serratorbita*	BSE	202, 204
Order Decapoda, Family Penaeidae	*Metapenaeopsis beebei* (Burkenroad, 1938)	BSE	198
	*Penaeus* sp. as *Pennaeus*	MCa	32
	*Penaeus* sp. as *Pennaeus*	MSa	32
	*Penaeus* sp. as *Pennaeus*	MPG	32, 208
	*Penaeus* sp. as *Pennaeus*	MPN	32
Order Decapoda, Family Pilumnidae	*Pilumnus pygmaeus* Boone, 1927	BSE	47, 81
Order Decapoda, Family Pinnotheridae	*Glassella costaricana* (Wicksten, 1982)	BEH	185
Order Decapoda, Family Porcellanidae	*Euceramus transversilineatus* (Lockington, 1878)	PMo	197
	*Euceramus transversilineatus* (Lockington, 1878)	BSE	90
	*Megalobrachium pacificum* Gore & Abele, 1973	BEH	197
	*Megalobrachium pacificum* Gore & Abele, 1973	PBl	87
	*Pachycheles biocellatus* (Lockington, 1878)	BSE	90
	*Pachycheles vicarius* Nobili, 1901	BSE	90
	*Petrolisthes agassizii* Faxon, 1893	PJu	197
	*Petrolisthes agassizii* Faxon, 1893	BSE	91
	*Petrolisthes armatus* (Gibbes, 1850)	Jun	197
	*Petrolisthes armatus* (Gibbes, 1850)	BSE	91
	*Petrolisthes artifrons* Haig, 1960	BSE	90
	*Petrolisthes edwardsii* (de Saussure, 1853)	Jun	197
	*Petrolisthes edwardsii* (de Saussure, 1853)	BSE	90
	*Petrolisthes edwardsii* (de Saussure, 1853)	PBl	90
	*Petrolisthes glasselli* Haig, 1957	BSE	90, 91
	*Petrolisthes haigae* Chace, 1962	Jun	197
	*Petrolisthes haigae* Chace, 1962	BSE	91
	*Petrolisthes haigae* Chace, 1962	PSE	197
	*Petrolisthes hians* Nobili, 1902	BSE	90, 91
	*Petrolisthes holotrichus* Nobili, 1901	BSE	90
	*Petrolisthes lewisi* Glassell, 1936 as *Petrolisthes lewisi austrinus*	BSE	90, 100
	*Petrolisthes lewisi* Glassell, 1936	PBl	197
	*Petrolisthes nobilii* Haig, 1960	BSE	90
	*Petrolisthes nobilii* Haig, 1960	PBl	197
	*Petrolisthes ortmanni* Nobili, 1901	BSE	90, 91
	*Petrolisthes ortmanni* Nobili, 1901	PBl	90
	*Petrolisthes platymerus* Haig, 1960	BSE	90, 101
Order Decapoda, Family Porcellanidae	*Petrolisthes platymerus* Haig, 1960	PBl	197
	*Petrolisthes polymitus* Glassell, 1937	PBl	90
	*Petrolisthes tonsorius* Haig, 1960	BSE	90
	*Petrolisthes tridentatus* Stimpson, 1859	BSE	90, 91
	*Pisidia magdalenensis* (Glassell, 1936)	Jun	197
	*Pisidia magdalenensis* (Glassell, 1936)	BSE	90, 91
	*Pisidia magdalenensis* (Glassell, 1936)	PBl	90
	*Polyonyx nitidus* Lockington, 1878	BJu	197
	*Porcellana cancrisocialis* Glassell, 1936	BSE	90, 91
	*Porcellana paguriconviva* Glassell, 1936	BSE	90, 91
Order Decapoda, Family Portunidae	*Achelous asper* (A. Milne-Edwards, 1861) as Portunus (Portunus) panamensis	BSE	81
	*Achelous tuberculatus* Stimpson, 1860 as Portunus (Acheolus) tuberculatus	BSE	81
	*Arenaeus mexicanus* (Gerstaecker, 1856)	IMu	81
	*Arenaeus mexicanus* (Gerstaecker, 1856)	BPG	81
	*Callinectes arcuatus* Ordway, 1863	BSE	81
	*Cronius ruber* (Lamarck, 1818)	BSE	81
	Portunus (Portunus) acuminatus (Stimpson, 1871)	**BSE^c^**	78, 81, 198
	Portunus (Portunus) asper (A. Milne-Edwards, 1861)	BSE	81
	Portunus (Portunus) asper (A. Milne-Edwards, 1861)	IMu	81
Order Decapoda, Family Pseudorhombilidae	*Lophoxanthus lamellipes* (Stimpson, 1860)	BSE	47
Order Decapoda, Family Sesarmidae	*Aratus pisonii* (H. Milne Edwards, 1837)	BSE	47
Order Decapoda, Family Sicyoniidae	*Sicyonia disdorsalis* (Burkenroad, 1934)	PNa	196
	*Sicyonia disedwardsi* (Burkenroad, 1934)	PMo	196
	*Sicyonia laevigata* Stimpson, 1871	BSE	196
	*Sicyonia laevigata* Stimpson, 1871	IMu	196
	*Sicyonia martini* Pérez-Farfante & Boothe, 1981	IDa	196
	*Sicyonia martini* Pérez-Farfante & Boothe, 1981	IMu	198
	*Sicyonia martini* Pérez-Farfante & Boothe, 1981	PNa	196
	*Sicyonia picta* Faxon, 1893	PNa	196
Order Decapoda, Family Solenoceridae	*Solenocera florea* Burkenroad, 1938	PBr	196
Order Decapoda, Family Trapeziidae	*Trapezia bidentata* (Forskål, 1775) as *Trapezia cymodoce ferruginea*	BSE	47
Order Decapoda, Family Upogebiidae	*Upogebia dawsoni* Williams, 1986	BPG	205
Order Decapoda, Family Xanthidae	*Cataleptodius taboganus* (Rathbun, 1912) as *Leptodius taboganus*	BSE	47
	*Cycloxanthops vittatus* (Stimpson, 1860)	BSE	47
	*Edwardsium lobipes* (Rathbun, 1898) as *Medaeus lobipes* in reference 81	BSE	81, 198
	*Heteractaea lunata* (Lucas, in H. Milne Edwards & Lucas, 1844)	BSE	47
	*Liomera cinctimanus* (White, 1847) as *Carpilodes cinctimanus*	BSE	47
	*Microcassiope xantusii* (Stimpson, 1871) as *Micropanope xantusii*	BSE	47
	*Paractaea sulcata* (Stimpson, 1860) as *Actaea sulcata*	BSE	47
Order Decapoda, Family Xanthidae	*Platyactaea dovii* (Stimpson, 1871) as *Actaea dovii*	BSE	47, 81
	*Williamstimpsonia stimpsoni* (Milne Edwards, 1879) as *Xanthodius stimpsoni*	BSE	47
	*Xanthodius sternberghii* Stimpson, 1859	BSE	47
Order Mysida, Family Mysidae	*Chlamydopleon banneri* (Bacescu, 1968) as *Bowmaniella banneri*	IMu	98
	Several species	IMu	171
Order Stomatopoda, Family Gonodactylidae	*Neogonodactylus bahiahondensis* (Schmitt, 1940)	PSE	195
	*Neogonodactylus bahiahondensis* (Schmitt, 1940)	IMu	195
	*Neogonodactylus costaricensis* (Manning & Reaka, 1979)	BSE	136
	*Neogonodactylus festae* (Nobili, 1901)	BSE	136
	*Neogonodactylus zacae* (Manning, 1972)	PMo	195
	*Neogonodactylus zacae* (Manning, 1972)	BSE	195
Order Stomatopoda, Family Nannosquillidae	*Nannosquilla canica* Manning & Reaka, 1979	PBl	137
	*Nannosquilla decemspinosa* (Rathbun, 1910) as *Lysiosquilla decemspinosa*	PBl	135
Order Stomatopoda, Family Pseudosquillidae	*Pseudosquillisma adiastalta* Manning, 1964	CSE	195
Order Stomatopoda, Family Squillidae	*Crenatosquilla oculinova* (Glassell, 1942)	CSE	195
	*Squilla biformis* Bigelow, 1891	CSE	129, 195
	*Squilla panamensis* Bigelow, 1891	CSE	195
Order Tanaidacea, Family Leptocheliidae	Several species	IMu	99
Order Tanaidacea, Family Parapseudidae	***Parapseudes latifrons* (Grube, 1864) as *Parapseudes pedispinis***	BSE	148
	***Parapseudes latifrons* (Grube, 1864) as *Parapseudes pedispinis***	PBl	148
	***Parapseudes latifrons* (Grube, 1864) as *Parapseudes pedispinous***	IMu	99
Order Tanaidacea, Family Tanaididae as Family Tanaidae	Several species	IMu	99
Class Maxillopoda, Subclass Copepoda, Family Acartiidae	**Acartia (Acartia) negligens Dana, 1849 as Acartia (Planktacartia) negligens**	IMu	187
Class Maxillopoda, Infraclass Cirripedia, Order Sessilia, Family Balanidae	*Amphibalanus inexpectatus* (Pilsbry, 1916) as *Balanus inexpectatus*	Jun	185
Order Sessilia, Family Chthamalidae	*Chthamalus panamensis* Pilsbry, 1916	Jun	185
	*Chthamalus panamensis* Pilsbry, 1916	CSE	27, 163
	***Microeuraphia imperatrix* (Pilsbry, 1916)**	CSE	27
Order Sessilia, Family Tetraclitidae	*Tetraclita stalactifera* (Lamarck, 1818)	Jun	185
Phylum BRYOZOA, Class Gymnolaemata, Order Cheilostomatida, Family Bugulidae	***Sessibugula translucens* Osburn, 1950**	BSE	156
Order Cheilostomatida, Family Exechonellidae	***Anexechona ancorata* Osburn, 1950**	BSE	156
Order Cheilostomatida, Family Phidoloporidae	***Rhynchozoon rostratum* (Busk, 1855)**	PBl	157
Order Cheilostomatida, Family Schizoporellidae	***Schizoporella inarmata* Hincks, 1884 as Schizoporella linearis var. inarmata**	BSE	157
Class Stenolaemata, Order Cyclostomatida, Family Crisiidae	***Crisia occidentalis* Trask, 1857**	BSE	158
Order Cyclostomatida, Family Lichenoporidae	***Disporella californica* (d’Orbigny, 1853)**	BSE	158
Order Cyclostomatida, Family Tubuliporidae	***Tubulipora pulchra* MacGillivray, 1885**	BSE	158
Order Cyclostomatida, Family Unassigned	***Diaperoforma californica* (d’Orbigny, 1853) as *Diaperoecia californica***	PBl	158
	*Petralia japonica* (Busk, 1884)	BSE	157
Phylum ECHINODERMATA, Class Asteroidea, Order Valvatida, Family Oreasteridae	*Pentaceraster cumingi* (Gray, 1840) as *Oreaster occidentalis*	BSE	28
Class Echinoidea, Order Camarodonta, Family Echinometridae	*Echinometra vanbrunti* A. Agassiz, 1863	Jun	185
Class Holothuroidea, Order Aspidochirotida, Family Holothuriidae	**Holothuria (Cystipus) rigida (Selenka, 1867) as *Fossothuria rigida***	PBl	63
	**Holothuria (Halodeima) kefersteinii (Selenka, 1867) as *Ludwigothuria kefersteini***	PBl	63
	Holothuria (Selenkothuria) lubrica Selenka, 1867	BSE	63
	Holothuria (Semperothuria) languens Selenka, 1867 as *Semperothuria languens*	PBl	63
	Holothuria (Thymiosycia) arenicola Semper, 1868 as *Brandtothuria arenicola*	BSE	63
	Holothuria (Thymiosycia) arenicola Semper, 1868 as *Brandtothuria arenicola*	PBl	63
	Holothuria (Thymiosycia) impatiens (Forskål, 1775) as *Brandtothuria impatiens*	BSE	63
	Holothuria (Thymiosycia) impatiens (Forskål, 1775) as *Brandtothuria impatiens*	PBl	63
Order Dendrochirotida, Family Cucumariidae	***Neocucumis veleronis* (Deichmann, 1941)**	**PBl^a^**	62
	***Pseudocnus californicus* (Semper, 1868) as *Cucumaria californica***	BSE	62
	***Pseudocnus dubiosus dubiosus* (Semper, 1868) as *Cucumaria dubiosa***	BSE	62
Order Dendrochirotida, Family Phyllophoridae	*Pentamera chierchiae* (Ludwig, 1887)	BSE	61, 62
	*Pentamera chierchiae* (Ludwig, 1887)	PBl	62
Order Dendrochirotida, Family Sclerodactylidae	***Afrocucumis ovulum* (Selenka, 1867) as *Euthyonidium ovulum***	BSE	61
	*Neothyone gibber* (Selenka, 1867)	BSE	62
	***Neothyone gibbosa* Deichmann, 1941**	PBl	62
Phylum CHORDATA, Subphyllum Tunicata, Class Ascidiacea, Order Aplousobranchia, Family Didemnidae	*Didemnum moseleyi* (Herdman, 1886) as *Didemnum mosseleyi*	IDa	154
	*Didemnum moseleyi* (Herdman, 1886) as *Didemnum mosseleyi*	BVi	154
	*Lissoclinum caulleryi* (Ritter & Forsyth, 1917)	IDa	154
	*Lissoclinum caulleryi* (Ritter & Forsyth, 1917)	BVi	154
Order Phlebobranchia, Family Ascidiidae	*Ascidia ceratodes* (Huntsman, 1912)	IDa	154
	*Ascidia ceratodes* (Huntsman, 1912)	BRo	154
	*Ascidia ceratodes* (Huntsman, 1912)	BVi	154
Order Aplousobranchia, Family Diazonidae	*Rhopalaea birkelandi* Tokioka, 1971	IDa	154
	*Rhopalaea birkelandi* Tokioka, 1971	BRo	154
	*Rhopalaea birkelandi* Tokioka, 1971	BVi	154
Order Stolidobranchia, Family Styelidae	*Eusynstyela tincta* (Van Name, 1902) as *Polyandrocarpa tincta*	IDa	154
	*Eusynstyela tincta* (Van Name, 1902) as *Polyandrocarpa tincta*	BVi	154
Subphylum Cepahalochordata, Class Leptocardii, Order Unassigned, Family Branchiostomatidae	*Branchiostoma californiense* Andrews, 1893	PJu	185
Subphylum Vertebrata, Superclass Pisces, Class Elasmobrabranchii, Order Myliobatiformes, Family Dasyatidae	*Dasyatis longa* (Garman, 1880) as *Dasyatis longus*	Cua	24, 178
Order Myliobatiformes, Family Urotrygonidae	*Urobatis pardalis* Del Moral-Flores, Angulo, López & Bussing, 2015	**IMu^b^**	64
Order Torpediniformes, Family Narcinidae	*Narcine entemedor* Jordan & Starks, 1895	Cua	40, 138, 178
Class Actinopteri, Order Anguilliformes, Family Muraenidae	*Echidna nocturna* (Cope, 1872)	Cua	156,178
Order Perciformes, Family Cirrhitidae	*Oxycirrhites typus* Bleeker, 1857	IMu	7
Order Perciformes, Family Gobiidae	*Chriolepis cuneata* Bussing, 1990	CSE	19
	*Chriolepis cuneata* Bussing, 1990	**IMu^a^**	19
	*Elacatinus digueti* (Pellegrin, 1901) as *Elacatious ioornatus* in reference 19	**IMu^a^**	19, 117
Order Perciformes, Family Haemulidae	*Microlepidotus brevipinnis* (Steindachner, 1869)	Cua	149
Order Perciformes, Family Labridae	*Thalassoma lucasanum* (Gill, 1862)	IMu	146
Order Perciformes, Family Labrisomidae	*Dialommus fuscus* (Gilbert, 1891)	IMu	88
	*Paraclinus monophthalmus* (Günther, 1861)	BSE	179
Order Perciformes, Family Opistognathidae	*Opistognathus fossoris* Bussing & Lavenberg, 2003	**IMu^a^**	21
Order Perciformes, Family Trpterygiidae	*Lepidonectes clarkhubbsi* Bussing, 1991	**BSE^b^**	20
	*Lepidonectes clarkhubbsi* Bussing, 1991	**CSE^b^**	20
	*Lepidonectes clarkhubbsi* Bussing, 1991	**IMu^a, b^**	20
Orden Syngnathiformes, Family Syngnathidae	*Bryx veleronis* Herald, 1940	IMu	88
Class Reptilia, Order Crocodylia, Family Crocodylidae	*Crocodylus acutus* (Cuvier, 1807)	MPG	128
	*Crocodylus acutus* (Cuvier, 1807)	MPN	140
Order Testudines, Family Cheloniidae	*Chelonia mydas agassizi* Bocourt, 1868	PNa	33, 34, 35, 65, 67, 115
	*Lepidochelys olivacea* (Eschscholtz, 1829)	PNc	1, 10, 25, 33, 35, 36, 37 48, 49, 70, 71, 74, 84, 89, 120, 121, 122, 123, 147, 148, 150, 153, 155, 164, 165, 166, 167, 168, 169, 175, 183, 190, 192, 193, 202
	*Lepidochelys olivacea* (Eschscholtz, 1829)	PNa	1, 34, 35, 65, 66, 67
Order Testudines, Family Dermochelyidae	*Dermochelys coriacea* (Vandelli, 1761)	PNa	34, 35, 65
Class Aves, Order Pelecaniformes, Family Fregatidae	*Fregata magnificens* Mathews, 1914	IBo	2
Order Pelecaniformes, Family Pelecanidae	*Pelecanus occidentalis* Linnaeus, 1766	PNa	3
Class Mammalia, Order Cetartiodactyla, InfraOrder Cetacea, SuperFamily Mysticeti, Family Balaenopteridae	*Megaptera novaeangliae* Borowski, 1781	SMa	139
SuperFamily Odontoceti, Family Delphinidae	*Globicephala macrorhynchus* Gray, 1846	IMu	145
	*Orcinus orca* Linnaeus, 1758	IMu	145
	*Orcinus orca* Linnaeus, 1758	SMa	139
	*Stenella attenuata graffmani* (Lönnberg, 1934)	Cua	145
	*Senella attenuata graffmani* (Lönnberg, 1934)	IMu	141, 142, 143, 144
	*Stenella attenuata* (Gray, 1846)	SMa	139
	*Tursiops truncatus* Montagu, 1821	SMa	139
SuperFamily Odontoceti, Family Kogiidae	*Kogia breviceps* (de Blainville, 1838)	IMu	145
SuperFamily Odontoceti, Family Physeteridae	*Physeter macrocephalus* Linnaeus, 1758 as *Physeter catodon*	PSE	177
